# The NF‐κB Signalling Pathway: Mechanisms, Consequences and Therapeutic Targets

**DOI:** 10.1111/jcmm.71229

**Published:** 2026-06-04

**Authors:** Bherouz Pourdad, Arash Pourdad

**Affiliations:** ^1^ Medical Biotechnology Department, Mashhad Medical School Mashhad University of Medical Sciences Mashhad Iran; ^2^ Department of Laboratory Sciences, School of Paramedical Sciences Mashhad University of Medical Sciences Mashhad Iran

**Keywords:** decoy, IKK, inflammation, IκB, JNK, liver, NF‐κB, oncogene

## Abstract

Nuclear Factor‐kappa B (NF‐κB) is a master transcriptional regulator orchestrating critical cellular processes, predominantly immunity and inflammation. However, its aberrant constitutive activation has emerged as a unifying pathogenetic hallmark across diverse malignancies, autoimmune disorders, and chronic inflammatory diseases. While the fundamental biology of canonical and non‐canonical NF‐κB signalling is well‐established, translating this extensive knowledge into clinically viable therapeutics remains severely hindered by dose‐limiting systemic toxicities and complex pharmacokinetic liabilities. This review critically evaluates the transition of NF‐κB from a basic biological paradigm to a highly challenging yet promising therapeutic target. After a streamlined synthesis of its signalling dynamics and pathological implications across various disease states, the core focus of this report shifts to an in‐depth analysis of next‐generation therapeutic interventions. We specifically dissect advanced molecular strategies, moving beyond conventional pharmacological inhibitors to emphasize nucleic acid‐based therapies, including decoy oligodeoxynucleotides (ODNs), peptide nucleic acids (PNAs), and locked nucleic acids (LNAs). Furthermore, we critically address current translational bottlenecks, highlighting the pivotal role of lipid nanoparticle (LNP) transporters and smart drug delivery systems in overcoming off‐target effects. By mapping these cutting‐edge modalities, this review underscores the critical necessity of transitioning from broad‐spectrum inhibition toward context‐specific, precision‐engineered modulation of the NF‐κB axis.

## 1 Introduction

### 1.1 Overview of NF‐κB and Its Fundamental Biological Roles

Initially identified as a regulator of B‐cell immunoglobulin expression, Nuclear Factor‐kappa B (NF‐κB) is now recognized as a ubiquitously expressed family of transcription factors central to cellular homeostasis. It coordinates a remarkable diversity of physiological processes—ranging from immunity and inflammation to cell survival, differentiation, and stress responses—enabling organisms to robustly adapt to environmental changes. While decades of research have firmly established the NF‐κB pathway as a paradigm for signal transduction and gene regulation, its highly pleiotropic nature presents a distinct conceptual and therapeutic challenge: understanding how a single signalling hub orchestrates such highly specific, context‐dependent outcomes. Addressing this complexity is essential for distinguishing its vital physiological roles from its pathological deregulation in disease states.

### 1.2 The Pathological Imperative of NF‐κB Dysregulation

Constitutive hyperactivation of NF‐κB represents a unifying pathogenic hallmark across a broad spectrum of chronic inflammatory syndromes, autoimmune disorders, and malignancies. Rather than serving merely as a downstream responder, aberrant NF‐κB signalling actively drives disease progression by perpetuating unresolved inflammatory microenvironments, conferring apoptotic evasion, and promoting malignant characteristics such as uncontrolled proliferation and metastasis. Consequently, the transition from transient physiological NF‐κB activation to sustained pathological signalling presents a critical cellular vulnerability that necessitates targeted, highly specific intervention.

### 1.3 Scope and Organization of the Review

While the molecular mechanics of NF‐κB are extensively documented, translating this fundamental knowledge into viable clinical strategies remains a profound translational bottleneck. Accordingly, to avoid redundancy with existing fundamental literature, this review provides only a streamlined overview of NF‐κB signalling dynamics and its environmental modulators. Crucially, the focal point of this report lies in critically evaluating the emerging landscape of NF‐κB‐targeted therapeutics. We dissect the mechanisms of current clinical modalities and heavily emphasize next‐generation interventions, particularly advanced nucleic acid therapeutics (Decoy ODNs, PNAs and LNAs). Furthermore, we outline inherent pharmacokinetic liabilities—such as systemic toxicity and poor bioavailability—and propose future directions focused on advanced targeted delivery systems, including Lipid Nanoparticles (LNPs). Ultimately, this review aims to provide a comprehensive roadmap for achieving precise, disease‐specific modulation of this master transcriptional regulator.

## 2 Materials and Methods

This article constitutes a comprehensive narrative review aimed at collecting, analysing, and synthesizing the existing knowledge regarding the Nuclear Factor kappaB (NF‐κB) signalling pathway, its activation mechanisms, pathophysiological roles, and therapeutic potential. As this is a narrative review and not a systematic review or meta‐analysis, it did not involve strict inclusion/exclusion criteria (such as PICO/PECO) for primary studies, interventions, comparisons, or outcomes, but rather provided an in‐depth exploration of the NF‐kappa B subject matter. As English is not the author's first language, an AI language model (ChatGPT, OpenAI) was used to assist with grammar and fluency. Figures and schematic illustrations were also created with the assistance of AI‐based design tools. The scientific content, interpretations, and conclusions remain solely the author's responsibility.

### 2.1 Search Strategy and Information Sources

A comprehensive literature search was conducted using major databases, including the National Center for Biotechnology Information (NCBI), PubMed, ScienceDirect and Google Scholar. To ensure extensive coverage of the literature, the search period was set from 1978 to 2025.

### 2.2 Search Keywords

The search keywords were utilized in various combinations and individually to query titles, abstracts, and full texts of the articles. The comprehensive set of keywords employed included: ‘NF‐kappaB, oncogene, inflammation, Inflammatory, IKK, JNK, Liver, immune system, Interleukin, IkappaB, MAP, MAPK, Tumor, Ikappa B kinase, TRAF, Oxidative stress, Toll‐like receptor, insulin, diabetes, pancreatitis, cancer, TNF, metabolic, ER Stress, leptin resistance, SIRT, Kidney Disease, NAFLD, Macrophage, Obesity, PDGF, growth factor, Apoptosis, hepatitis, cytokines, decoy, oligonucleotides, nucleic acids, nanoparticles’.

### 2.3 Screening and Selection Process

A total of 823 articles related to the specified keywords were initially identified. The article selection process involved two main stages:

1. Initial screening: in the first stage, all identified articles were screened independently by two authors based on the title, and for some, the title and abstract to ensure their relevance to the NF‐kappaB signalling pathway, its consequences, or therapeutic targets.
2. Secondary screening and content review: in this phase, the abstracts and results, and in several instances, the full text of the initially accepted articles were studied in depth. The two authors independently assessed the content, selecting articles that provided crucial and relevant information aligned with the scope of this review (mechanisms, pathophysiological role, and therapeutic approaches). Any potential discrepancies in selection were resolved through discussion and consensus.

### 2.4 Data Utilization

Following the independent screening process, a total of 353 articles out of the 823 initial search results were selected, analyzed, and synthesized to construct this review. The data from these articles were utilized to detail the structure of the NF‐kappa B family, signaling pathways, involvement in diseases, and therapeutic strategies targeting NF‐kappaB.

### 2.5 Population, Intervention, Comparison and Outcome (PICO)

As stated, this is not a systematic review, but rather aims to provide a comprehensive overview, review of mechanisms, and therapeutic outlook for NF‐kappaB in biology and medicine. Consequently, the PICO criteria, which are mandatory for systematic reviews and meta‐analyses, were not applicable and were not applied in this study.

## 3 The NF‐κB Family of Transcription Factors

### 3.1 Subunit Composition and Dimerization Complexity

The mammalian NF‐κB family comprises five structurally related transcription factors classified into two subfamilies: the NF‐κB proteins (NF‐κB1/p50 and NF‐κB2/p52, processed from p105 and p100 precursors, respectively) and the Rel proteins, which inherently possess transactivation domains (RelA/p65, RelB and c‐Rel) [1, 2, 3]. Upon activation by diverse pathogenic or inflammatory stimuli, these latent cytoplasmic factors translocate to the nucleus, where they critically orchestrate innate and adaptive immune cascades, including targeted lymphocyte proliferation and macrophage activation [1, 4, 5, 6, 7, 8].

A defining hallmark of NF‐κB signalling is its profound combinatorial complexity, achieved through the formation of up to 15 distinct homo‐ and heterodimeric complexes [9, 10]. This dimerization intricately dictates DNA‐binding specificity and transcriptional outcomes. For instance, p50 or p52 homodimers, lacking transactivation domains, generally function as transcriptional repressors; however, their heterodimerization with Rel members (e.g., p50/p65 or p52/RelB) robustly converts them into potent transcriptional activators [3]. This structural diversity enables highly tissue‐ and context‐dependent transcriptional responses to external stimuli [7].

Crucially, from a translational perspective, this combinatorial complexity underscores the necessity for precision therapeutics. Moving beyond broad‐spectrum, pan‐NF‐κB inhibition—which is often limited by systemic toxicity—future clinical strategies must focus on selectively targeting specific, disease‐driving dimeric complexes to achieve maximum efficacy with minimal off‐target effects [10].

### 3.2 Structural Architecture and Functional Domains

The defining structural hallmark of all NF‐κB proteins is the highly conserved N‐terminal Rel Homology Domain (RHD, ∼/sim ∼300 amino acids). This critical domain physically orchestrates DNA binding, subunit dimerization, nuclear localization, and interaction with IκB inhibitory proteins [5]. Functionally, the family bifurcates based on C‐terminal architecture: RelA (p65), c‐Rel and RelB possess transactivation domains (TADs) essential for driving gene expression. In contrast, p50 and p52 naturally lack TADs. Consequently, their respective homodimers act as intrinsic transcriptional repressors, requiring heterodimerization with TAD‐containing Rel members to acquire transactivating capabilities [11].

**FIGURE 1:**
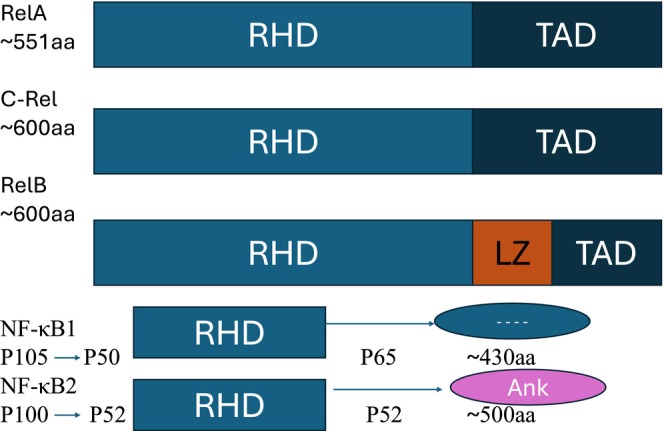
Structural topography of the NF‐κB family. Schematic representation highlighting the conserved N‐terminal Rel Homology Domain (RHD) across all members and the C‐terminal Transactivation Domains (TAD) specific to Rel proteins. The p105 and p100 precursors are characterized by C‐terminal Ankyrin (Ank) repeats, which undergo proteolytic processing to yield the mature p50 and p52 subunits, respectively. aa: approximate amino acid length; LZ: Leucine Zipper.

Beyond basal structural interactions, the DNA‐binding affinity and ultimate transcriptional output of these dimers are strictly governed by diverse post‐translational modifications (PTMs). The specific topographical organization of these domains—including the unique Leucine Zipper (LZ) motif in RelB and the inhibitory Ankyrin repeats in the precursors—is delineated in Figure 1.

### 3.3 Physiological Roles of Distinct NF‐κB Dimers

The combinatorial assembly of NF‐κB subunits generates extensive transcriptional diversity. The prototypical and most ubiquitous active form is the p50: p65 heterodimer, which binds with high affinity to consensus κ\kappaκB sequences (e.g., 5′‐GGGPuNNPyPyCC‐3′) to drive robust gene induction [11, 12, 13]. Upon binding, the p65 transactivation domain (TAD) physically recruits basal transcription machinery (such as TBP and TFIIB) and auxiliary co‐activators to initiate transcription [13]. Conversely, TAD‐deficient homodimers (e.g., p50: p50) generally act as intrinsic repressors, though they can atypically drive specific targets like IL‐1‐induced collagenase depending on the cellular context [5, 14]. Higher‐order complex formations (e.g., with Bcl‐3) and diverse post‐translational modifications (PTMs) further fine‐tune the DNA‐binding affinity and spatiotemporal specificity of these dimers [9, 15].

In unstimulated cells, NF‐κB dimers are tightly sequestered in the cytoplasm by IκB inhibitors or the C‐terminal Ankyrin repeats of p100/p105 precursors, which also utilize their death domains (DD) as signalling adaptors [2, 6, 16]. The activation cascades consistently converge on the IKK complex (IKKα/IKKβ). Upon stimulation by diverse triggers—including pathogens, cytokines (e.g., TNF‐α, IL‐1), or oxidative/ER stress—IKK phosphorylates IκB proteins, targeting them for 26S proteasomal degradation [11, 17, 18, 19, 20]. Similarly, IKK drives the partial proteolysis of p100 into mature p52, halting at the glycine‐rich region (GRR) to liberate active dimers for nuclear translocation [11, 17].

Functioning as the master regulator of both innate and adaptive immunity, NF‐κB orchestrates the expression of hundreds of pro‐inflammatory mediators and dictates T cell activation and differentiation [12, 13, 21, 22]. Notably, NF‐κB signalling critically intersects with inflammasome pathways (via NLRs), driving the release of IL‐1β and IL‐18 through caspase‐1 activation [23, 24]. This crosstalk is pathogenically significant in liver diseases; for instance, endosomal TLR9 activation by CpG DNA initiates MyD88/NF‐κB signalling in Kupffer cells, heavily contributing to hepatic injury, steatosis, and fibrosis in NASH models [17, 25]. Knockout studies corroborate the non‐redundant, highly specialized roles of distinct NF‐κB members in host defence [7]. Given that persistent nuclear localization of p65 is a universal hallmark of chronic inflammatory syndromes [8, 16, 17, 20, 26], mapping these intricate dimer‐specific functions is imperative for designing precision therapeutics [22]. The divergent biological functions of the NF‐κB family members are consolidated in Table 1.

**TABLE 1:** Core functional and phenotypic distinctions among NF‐κB family members.

NF‐κB member	Pathway association and core functions	Phenotypic hallmark (from knockout models)	Primary IκB interactors	References
NF‐κB1 (p50/p105)	Canonical pathway: acts as a dual‐function transcriptional modulator (repressor/activator). Governs innate immunity, inflammation, and B‐cell responses	Impaired B‐cell proliferation and humoral immunity; multi‐organ inflammation	IκBα, IκBβ	[2, 18, 27, 28]
NF‐κB2 (p52/p100)	Non‐canonical pathway: critical for secondary lymphoid organogenesis, B‐cell maturation, and osteoclastogenesis	Defective B‐cell maturation and complete absence of lymph nodes and Peyer's patches	Primarily regulated by precursor p100 processing	[29]
p65 (RelA)	Canonical pathway: master transactivator subunit. Regulates a vast network of genes involved in anti‐apoptosis, inflammation, and cell survival	Embryonic lethality due to massive fetal liver apoptosis	IκBα, IκBβ, IκBε	[18, 27, 28, 30, 31, 32, 33]
RelB	Non‐canonical pathway: key effector regulating dendritic cell homeostasis, lymphoid architecture, and adaptive immunity	Severe multi‐organ inflammation, myeloid hyperplasia, and dysfunctional adaptive immunity	IκBα, IκBβ (regulated by p100)	[8, 27, 29, 31]
c‐Rel	Canonical pathway: predominantly involved in adaptive immunity; essential for lymphocyte activation, proliferation, and differentiation. Implicated in lymphoid malignancies	Severely impaired T‐ and B‐cell proliferation and defective humoral responses	IκBβ, IκBε	[29, 31]

### 3.4 Critical Summary: The Translational Imperative of NF‐κB Complexity

The structural and combinatorial diversity of the NF‐κB family presents a formidable double‐edged sword in pharmacological intervention. While the intricate dimerization networks and context‐dependent post‐translational modifications (PTMs) grant exquisite spatiotemporal control over immune homeostasis, this very complexity constitutes a major translational bottleneck. Historically, therapeutic strategies have relied on broad‐spectrum, pan‐NF‐κB inhibition, which invariably triggers prohibitive systemic toxicities and severe immunosuppression by disrupting basal physiological functions. The critical insight derived from the structural and phenotypic distinctions of these subunits is that pathological states—such as chronic inflammation, fibrosis or oncogenesis—are frequently driven by specific hyperactive complexes, notably the persistent nuclear localization of the p50: p65 heterodimer, rather than a universal family‐wide dysregulation. Therefore, the future of NF‐κB‐targeted therapy hinges on a critical paradigm shift: abandoning blunt‐force systemic blockade in favour of precision medicine. Moving forward, overcoming this translational hurdle necessitates exploiting nuanced structural vulnerabilities, selectively targeting disease‐specific dimer interfaces, and identifying targeted delivery systems capable of uncoupling pathological NF‐κB signalling from essential host defence mechanisms.

## 4 Regulatory Dynamics of NF‐κB Activation and Inhibition

NF‐κB functions as a rapid, translation‐independent responder to diverse pathogenic and cytokine stimuli, orchestrating essential immune and acute‐phase defenses [34, 35, 36]. While IκB degradation initiates signalling, precise spatiotemporal gene expression is achieved through a complex interplay of cell‐type‐specific receptor profiles, IKK/IκB isotype hierarchies, and diverse DNA‐binding motifs [7]. Furthermore, the pathway is intricately fine‐tuned by a network of post‐translational modifications (PTMs)—including phosphorylation, ubiquitination, and sumoylation—targeting both upstream kinases and the NF‐κB subunits themselves [34]. Transgenic models confirm that constitutive dysregulation of these regulatory layers drives the pathogenesis of malignancies, metabolic syndromes, and organ failure [34] Critically, this elaborate multi‐layered regulation highlights why broad‐spectrum NF‐κB inhibition often fails clinically; effective therapeutic interventions must pivot toward targeting context‐dependent PTMs or specific regulatory nodes rather than universally blunting the cascade.

### 4.1 Endogenous Inhibitory Mechanisms: IκB and Precursor Proteins

In quiescent cells, NF‐κB dimers are dynamically sequestered in the cytoplasm through non‐covalent interactions with IκB family members (e.g., IκBα, IκBβ, IκBϵ and Bcl‐3) [36, 37] and the unprocessed precursors p105 and p100 [38, 39]. This cytosolic retention is mediated by multiple ankyrin repeats—present in the central domain of classical IκBs and the C‐termini of precursors—which physically engage the Rel Homology Domain (RHD) and obscure the nuclear localization signal (NLS) of NF‐κB subunits [36, 37, 38, 39]. The modular architecture of classical IκBs intricately couples signal sensing to turnover: the N‐terminus harbours key phosphorylation sites for stimulus‐induced proteasomal clearance, while the C‐terminal PEST domain regulates basal half‐life. Similarly, the generation of mature subunits (e.g., p50 from p105) dictates an ATP‐dependent ubiquitin‐proteasome processing step [38, 39]. Critically, the structural diversity of these endogenous inhibitors functions as a sophisticated rheostat, fine‐tuning the temporal dynamics of NF‐κB responses; exploiting these distinct IκB‐NF‐κB interfaces presents a compelling, albeit challenging, frontier for selective pharmacological intervention.

Mechanistically, classical IκB proteins (IκBα, IκBβ and IκBϵ) inhibit NF‐κB by masking the Nuclear Localization Signal (NLS) of specific subunits (e.g., p65) via ARD‐RHD interactions. However, they are not merely static anchors; for instance, IκBα possesses a Nuclear Export Signal (NES) that drives continuous nucleocytoplasmic shuttling, maintaining a dynamic basal equilibrium until signal‐induced degradation permits robust nuclear translocation [5, 15, 40]. Within this classical family, kinetic and binding disparities dictate distinct transcriptional profiles: IκBα typically mediates rapid, transient responses and binds diverse complexes with comparable affinity (without impeding p50 homodimer DNA‐binding) [7, 28]. Conversely, IκBβ governs sustained activation and exhibits higher affinity for p50‐p65 heterodimers, while IκBϵ—which undergoes analogous signal‐induced hyperphosphorylation—acts as a predominant, potent inhibitor for RelA in specific cellular contexts [7, 31, 41].

**FIGURE 2:**
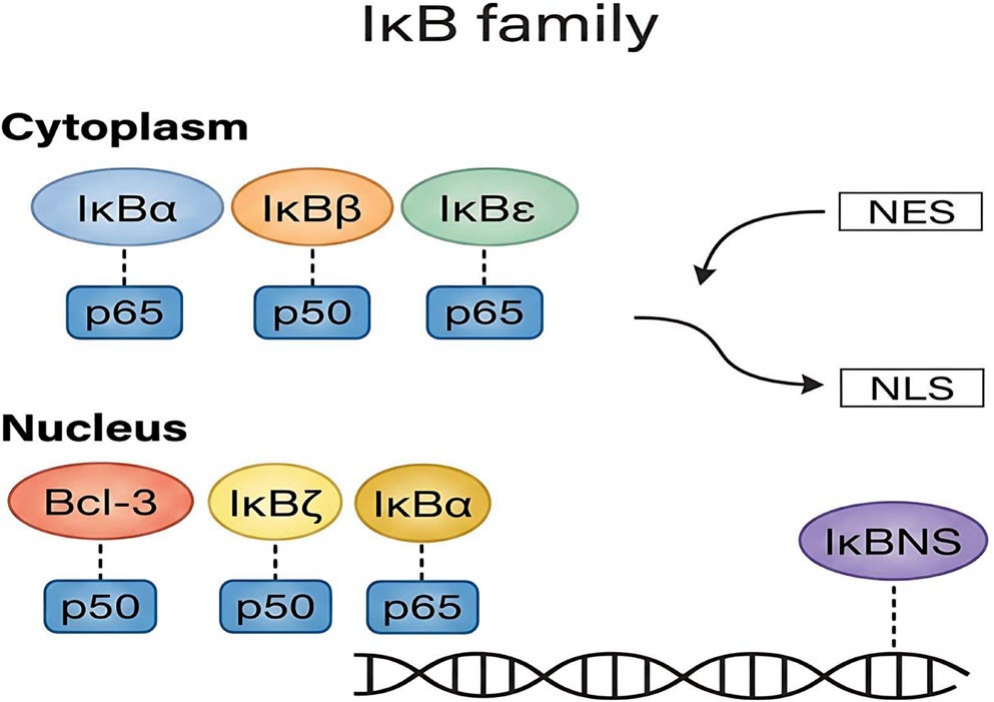
Subcellular compartmentalization and dynamic regulatory modalities of the IκB family. The schematic illustrates the functional dichotomy of IκB proteins across distinct cellular compartments. In the cytoplasm, classical IκB members (IκBα, IκBβ, and IκBε) act as primary gatekeepers, selectively sequestering NF‐κB dimers (e.g., p65 and p50) by masking their Nuclear Localization Signals (NLS); concurrent regulation via intrinsic Nuclear Export Signals (NES) maintains basal homeostasis through continuous nucleocytoplasmic shuttling. In the nucleus, atypical IκB members (Bcl‐3, IκBζ and IκBNS) function as sophisticated chromatin‐associated rheostats, directly engaging DNA‐bound NF‐κB complexes to bidirectionally modulate transcriptional outputs. Additionally, precursor proteins (p105 and p100) exhibit a dual functionality, serving as both NF‐κB subunits and intrinsic IκB‐like inhibitors. Note the presence of IκBα within the nuclear compartment, highlighting its critical role in terminating transcription via an autoregulatory negative feedback loop. NES, Nuclear Export Signal; NLS, Nuclear Localization Signal.

Beyond cytoplasmic sequestration, atypical IκB proteins (such as IκBζ and Bcl‐3) orchestrate complex nuclear modulation. Unlike their classical counterparts, these nuclear regulators often contain intrinsic Transactivation Domains (TADs). By predominantly engaging p50 and p52 homodimers (which naturally lack TADs) on DNA, they function as bifunctional rheostats—capable of acting either as potent transcriptional co‐activators or repressors, entirely contingent upon the specific dimer configuration and cellular microenvironment [15, 40, 41, 42]. This paradigm shift—redefining IκBs from exclusive repressors to versatile, tissue‐specific modulators [15]—is pivotal for understanding processes from B‐cell survival to leukemogenesis [15, 40]. Crucially, the non‐redundant and selective inhibitory profiles of these diverse IκB molecules provide an attractive rationale for developing highly specialized, precision therapeutics aimed at uncoupling pathological NF‐κB activity from physiological immunity [7] (Figure 2).

### 4.2 Endogenous Activation Mechanisms: The IKK Complex, NIK and TRAF Axes

The central hub driving NF‐κB activation is the IκB Kinase (IKK) holocomplex, comprising the catalytic subunits IKKα and IKKβ [43, 44, 45], the regulatory scaffold IKKγ/NEMO [46], and, in specific immune contexts, the inducible kinase IKKi [47]. In the canonical pathway, IKKβ acts as the dominant catalytic driver and a critical convergence point for upstream pro‐inflammatory signals, including Pattern Recognition Receptors (PRRs) and kinases such as MEKK and NAK [7, 43, 48, 49, 50]. Upon stimulus‐dependent phosphorylation, IKKβ catalyses the specific phosphorylation of classical IκBs, dictating their rapid proteasomal clearance and unleashing NF‐κB dimers to govern diverse cellular programs [45, 46, 51, 52, 53]. The physiological indispensability of IKKβ is profoundly illustrated in targeted ablation models, which exhibit lethal hepatocyte apoptosis and defective signalling responses to TNF‐α and IL‐1 [54]. Furthermore, IKK components are not isolated entities; they actively engage in signal integration, orchestrating extensive crosstalk with parallel networks like the p53, MAPK and IRF pathways [53].

In stark contrast, the non‐canonical pathway operates independently of IKKβ/NEMO, relying exclusively on a tightly regulated NIK‐IKKα axis [55]. Under homeostatic conditions, NF‐κB Inducing Kinase (NIK) is constitutively repressed via TRAF3/cIAP‐mediated ubiquitination and degradation. Activation of specific TNF‐receptor superfamilies induces TRAF3 degradation, stabilizing NIK. Accumulated NIK subsequently functions as both an adaptor and a kinase, recruiting and phosphorylating IKKα, which in turn catalyzes the ATP‐dependent proteolytic processing of the p100 precursor into mature p52 [55, 56]. This sophisticated processing machinery permits the nuclear translocation of atypical heterodimers (predominantly p52/RelB) and is absolutely imperative for B‐cell maturation, secondary lymphoid organogenesis, and the regulation of cell survival [1, 55, 56, 57].

This functional dichotomy between the IKK catalytic subunits underscores their non‐redundant roles. While both can phosphorylate IκB, IKKβ is the substantially more efficient kinase in vivo, driving the majority of canonical signalling in response to pro‐inflammatory stimuli across diverse cell types, including synoviocytes and lymphocytes [7, 58]. Its activity is tightly controlled via a C‐terminal autophosphorylation cluster, which functions as an autoinhibitory feedback loop to terminate the inflammatory cascade [59]. In contrast, IKKα's dominant role lies in the non‐canonical pathway, specifically mediating the phosphorylation of the p100 precursor [11]. The critical nature of this function is highlighted by IKKα knockout models, which exhibit profound defects in morphogenesis, most notably a failure in epidermal stratification and keratinocyte differentiation [60, 61].

The therapeutic implications of targeting this axis are complex, as revealed in preclinical models. While inducible, long‐term NF‐κB inhibition via an IκBα super‐repressor is well‐tolerated in adult mice without overt hepatotoxicity, it critically compromises host defense, rendering the animals highly susceptible to systemic bacterial challenges like *Listeria monocytogenes* [62]. Beyond immunity, the IKKβ‐NF‐κB axis is a crucial nexus for immunometabolic signalling. In hepatic tissue, aberrant IKKβ activity links lipid accumulation to subacute inflammation, driving both local and systemic insulin resistance [61]. Furthermore, this pathway influences cholesterol biosynthesis through the mevalonate pathway, positioning chronic IKK‐mediated inflammation as a key contributor to the pathophysiology of Metabolic Dysfunction‐Associated Fatty Liver Disease (MAFLD) and associated cardiovascular risks [63].

Concurrent with NF‐κB, inflammatory cytokines and stress stimuli orchestrate cellular fate via the Mitogen‐Activated Protein Kinase (MAPK) cascades, encompassing the ERK1/2, p38 and JNK pathways [64, 65]. Upstream activation of the NF‐κB axis exhibits distinct, receptor‐specific adaptor usage: TLRs, IL‐1 and IL‐18 depend on IRAK/TRAF6 complexes, whereas TNF signalling utilizes TRADD and RIP/TRAF2 to engage upstream kinases like NIK, ultimately culminating in IκB proteasomal degradation and NF‐κB nuclear translocation [5, 50]. However, the reliance on downstream catalytic subunits is highly context‐dependent, particularly in pathological settings. For instance, in primary fibroblast‐like synoviocytes (FLS) from rheumatoid and osteoarthritis patients, TNF‐α and IL‐1‐induced inflammatory cascades are strictly mediated by IKKβ. Studies utilizing dominant‐negative mutants unequivocally demonstrate that IKKβ, rather than IKKα, is the indispensable subunit required for endogenous IκBα degradation and the subsequent pathogenic upregulation of IL‐6, IL‐8, ICAM‐1 and Collagenase‐1 in these cells [7].

NF‐κB orchestrates a robust cellular survival program by transcriptionally upregulating anti‐apoptotic effectors (e.g., cIAPs, c‐FLIP, Bcl‐xL) that antagonize death receptor and mitochondrial apoptotic pathways [66]. Concurrently, NF‐κB mitigates TNF‐induced JNK hyperactivation [67, 68] through the induction of XIAP, GADD45 and the mitochondrial antioxidant SOD2, thereby inextricably linking NF‐κB activation to ROS suppression [11]. The in vivo indispensability of this survival axis is profoundly evident during embryogenesis; mice lacking p65, IKKβ, or IKKγ experience lethal hepatocyte apoptosis at mid‐gestation, whereas IKKα deficiency does not compromise liver survival [58, 69]. This reliance on IKKβ extends to mature hepatocytes, where it acts as the master kinase orchestrating both IκB degradation and the direct phosphorylation of p65 transactivation domains, shielding cells from TNF‐mediated cytotoxicity [69, 70]. Furthermore, a reciprocal regulatory crosstalk exists between the IKK/NF‐κB axis and metabolic sensors such as AMPK. While AMPK signalling broadly attenuates NF‐κB‐driven inflammation across various immune cells [71, 72]. Inflammatory cascades inversely suppress AMPK. Notably, TNF signalling—potentially via the IKK/NF‐κB axis—promotes AMPK inactivation through the upregulation of PP2C in metabolic tissues [63, 73, 74]. IKK‐mediated pathways further integrate inflammation with metabolic reprogramming by modulating HMGCR phosphorylation and HMGCS1 expression [59].

**FIGURE 3:**
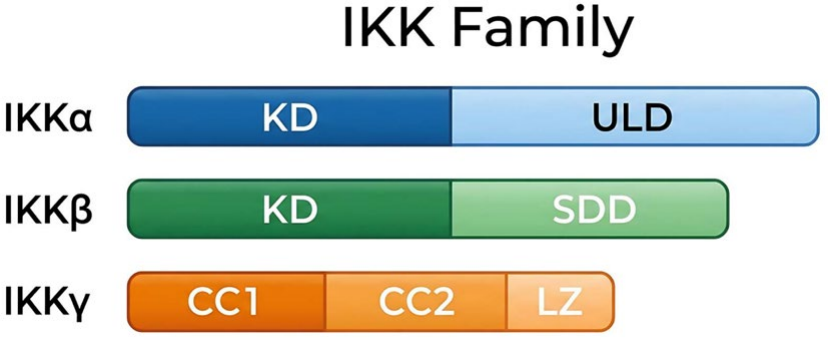
Structural architecture of the IKK family members. The catalytic subunits, IKKα and IKKβ, share an N‐terminal Kinase Domain (KD), succeeded by a Ubiquitin‐like Domain (ULD) in IKKα and a Scaffold/Dimerization Domain (SDD) in IKKβ, the latter being critical for complex assembly and signalling. Conversely, the non‐catalytic regulatory scaffold, IKKγ (NEMO), facilitates complex stability and signal transduction via its Coiled‐Coil (CC1 and CC2) and Leucine Zipper (LZ) domains.

Beyond the canonical proteasome‐mediated degradation of IκB and the subsequent release of DNA‐binding dimers, NF‐κB is subject to intricate downstream regulatory layers. These fine‐tuning mechanisms include promoter‐specific dimer exchange and complex post‐translational modifications (PTMs) of the transactivating p65 subunit, encompassing phosphorylation, acetylation, ubiquitination, and prolyl isomerization [75]. Central to orchestrating the upstream activation of these cascades is the IKK complex (Figure 3).

### 4.3 The NF‐κB Target Gene A20 (TNFAIP3) as a Negative Feedback Regulator

A20 (also known as TNFAIP3) is a critical NF‐κB‐inducible negative feedback regulator, initially characterized as a TNF‐responsive gene in endothelial cells [76]. Structurally, its C‐terminal domain harbours seven zinc finger (ZnF) motifs that mediate A20 dimerization and orchestrate essential protein–protein interactions to suppress NF‐κB activation and inhibit apoptosis. Operating primarily upstream of the IKK complex, A20 acts as a molecular brake to terminate signalling cascades through multifaceted mechanisms [63]. A defining feature of its inhibitory function involves the modulation of linear polyubiquitination, a critical post‐translational modification in TNF‐induced signalling. Specifically, A20 binds to linear ubiquitin chains via its ZnF7 domain, thereby antagonizing the Linear Ubiquitin Assembly Complex (LUBAC) and sterically hindering the essential interaction between LUBAC and NEMO [77]. Underscoring its indispensable role in immune homeostasis, genetic variants of *TNFAIP3* are established susceptibility loci for a broad spectrum of autoimmune and chronic inflammatory syndromes (including rheumatoid arthritis, Crohn's disease and systemic sclerosis), while A20 insufficiency is a recognized driver in the pathogenesis of human B‐cell lymphomas [48, 76].

### 4.4 Critical Summary: The Pharmacological Conundrum of NF‐κB Regulatory Networks

The regulatory architecture of the NF‐κB pathway functions not as a simple linear cascade, but rather as an exquisitely calibrated, multidimensional rheostat. The intricate spatial dichotomy between cytosolic sequestration by classical IκBs and the context‐dependent, chromatin‐associated modulation by atypical isoforms, alongside the strictly non‐redundant roles of the IKKα and IKKβ catalytic subunits, presents a profound pharmacological conundrum. Historically, translational efforts have faltered by treating this highly integrated network as a binary on/off switch. Deploying blunt, upstream pan‐inhibitors invariably dismantles critical physiological safety nets, precipitating prohibitive on‐target toxicities—such as catastrophic hepatocyte apoptosis, metabolic dysregulation and severe immunosuppression. Furthermore, the existence of robust endogenous fail‐safes, particularly the A20‐mediated negative feedback loop, underscores the evolutionary imperative of precise signal termination over absolute ablation. Consequently, realizing the clinical potential of NF‐κB modulation mandates a fundamental departure from global signalling blockade. The next frontier in precision therapeutics relies on exploiting these inherent regulatory nuances: engineering modalities that selectively intercept disease‐specific post‐translational modifications (PTMs), allosterically modulate distinct IKK conformers, or therapeutically mimic endogenous molecular brakes like A20, thereby neutralizing pathological hyperactivation while strictly preserving essential host defence and metabolic homeostasis.

## 5 The Canonical and Non‐Canonical NF‐κB Signalling Pathways

NF‐κB activation is orchestrated through two principal signalling cascades, distinguished by their mechanism of releasing active, DNA‐binding dimers [33]. The canonical pathway relies on the signal‐induced, proteasomal degradation of IκB inhibitors, whereas the non‐canonical pathway involves the proteolytic processing of precursor proteins p100 and p105 into their mature p52 and p50 forms. Superimposed on this foundational framework is an additional layer of regulatory complexity conferred by post‐translational modifications (PTMs)—including phosphorylation, acetylation and ubiquitination—which fine‐tune critical functions such as nuclear translocation, DNA binding and transactivation potential [24].

The canonical pathway, the principal route for responses to pro‐inflammatory stimuli like TNF‐α, IL‐1 and Toll‐like Receptor (TLR) agonists, hinges upon the activation of the IKKβ and NEMO (IKKγ) subunits [57, 75]. This leads to the rapid, phosphorylation‐dependent ubiquitination and proteasomal degradation of IκBα inhibitors, resulting in a rapid yet transient nuclear translocation of NF‐κB dimers, primarily p50/p65 [78]. In contrast, the non‐canonical (or alternative) pathway is governed by the inducible processing of the p100 precursor to its p52 active form, a process dependent on IKKα, not IKKβ [19, 79]. This cascade operates with slow and sustained kinetics, reflecting its integral role in more specialized, long‐term processes such as lymphoid organogenesis and B‐cell survival and maturation [75, 78, 79, 80]. A third, atypical pathway activated by genotoxic stress has also been described [75].

The pronounced kinetic dichotomy between the rapid/transient canonical and the slow/sustained non‐canonical pathways underscores their distinct physiological mandates: the former is tailored for acute inflammatory and innate immune responses, while the latter governs precise developmental and homeostatic functions [57, 81]. This distinction is of profound therapeutic relevance, as the development of pathway‐selective inhibitors is paramount to targeting pathological NF‐κB activity in chronic diseases without disrupting essential physiological processes.

### 5.1 Canonical NF‐κB Pathway: Activation Dynamics and Functional Consequences

In the basal state, classic NF‐κB dimers—predominantly NF‐κB1p50‐RelA and NF‐κB1p50‐c‐Rel—are constitutively sequestered in the cytoplasm via binding to IκB proteins, primarily the prototype IκBα [81]. This spatial inhibition is rapidly relieved upon exposure to a broad spectrum of proinflammatory signals, including TLR ligands, TNF‐α, IL‐1 and various cellular stressors [81]. The central signalling node of this canonical cascade is the IKK holocomplex, wherein IKKβ and NEMO/IKKγ are indispensable for activation, rendering IKKα largely redundant in this specific context [82, 83]. Activation culminates in IKKβ‐mediated phosphorylation of IκBα (at Ser32 and Ser36), triggering its rapid polyubiquitination and subsequent proteasomal degradation [82, 83].

The destruction of IκBα unmasks the nuclear localization signals (NLS) on the liberated heterodimers (typically p65/p50), facilitating their nuclear translocation to orchestrate a rapid, albeit transient, transcriptional cascade [82, 83]. This response not only drives the robust expression of critical inflammatory mediators (e.g., TNF‐α, IL‐1β, IL‐6, COX‐2, iNOS and chemokines) but also induces intrinsic negative regulators such as IκBα, A20 and p105, thereby establishing an autoregulatory feedback loop to resolve the signalling [82, 83]. From a clinical standpoint, while dampening this cascade is highly desirable, systemic IKKβ inhibition presents significant translational challenges; notably, the paradoxical risk of exacerbated IL‐1β production and severe cytokine‐driven neutrophilia during bacterial infections [82, 83].

While complete NF‐κB blockade is notoriously immunosuppressive, selective pharmacological IKKβ inhibition strategically preserves essential innate immunity [9]. This clinically viable, partial suppression is attributed to titratable enzyme kinetics, the sparing of the IKKα‐driven non‐canonical axis, and the existence of compensatory IKK‐independent activation pathways [9]. Consequently, targeted IKKβ antagonism avoids broad immunological paralysis and offers paradoxical therapeutic benefits, such as mitigating radiotherapy‐ or chemotherapy‐induced neutropenia [9]. Upstream of this regulatory node, the TAK1 kinase acts as a critical signal transducer, integrating inputs from diverse receptors (including TLRs, IL‐1R, TNFR and antigen receptors) to engage the IKK complex [20]. Beyond classic inflammatory responses, the ensuing canonical signalling drives a robust transcriptomic program—encompassing adhesion molecules (e.g., VCAM‐1, ICAM‐1, E‐selectin), angiogenic factors (VEGF), and matrix metalloproteinases (MMPs)—that fundamentally fuels cell survival, angiogenesis, and tumour metastasis [20].

### 5.2 Non‐Canonical NF‐κB Pathway: Components, Activation and Functional Consequences

Activated by specific TNF superfamily members (e.g., BAFF, CD40L, RANKL and Lymphotoxin) rather than classical inflammatory cues, the non‐canonical pathway operates through a central NF‐κB‐inducing kinase (NIK)‐IKKα axis [20]. This cascade exclusively drives the proteolytic processing of the p100 precursor into p52, generating transcriptionally active RelB/p52 heterodimers [57, 78]. Exhibiting characteristically slow and persistent kinetics, this signalling branch governs a distinct transcriptional program encompassing essential chemokines (CCL19, CCL21 and CXCL13) and adhesion molecules (VCAM1, ICAM1 and MADCAM1) [82, 84]. Physiologically, it is indispensable for B and T lymphocyte maturation, survival, and the structural organogenesis of secondary lymphoid tissues [20, 57, 79]. Pathologically, beyond immune dysregulation, aberrant non‐canonical signalling—particularly in distinct cell types like hepatocytes and thymic epithelial cells—is emerging as a critical driver in diverse hepatic pathologies, including NAFLD, ALD, autoimmune and viral liver diseases [78, 85, 86, 87].

Initiated by specific TNFR superfamily members harbouring intracellular TRAF‐binding motifs (e.g., LTβR, BAFFR, CD40, RANK, Fn14 and OX40), the non‐canonical cascade relies on the indispensable NIK‐IKKα axis, functioning entirely independently of IKKβ and NEMO [56, 80, 84, 88]. Under basal conditions, the central kinase NIK is strictly repressed through constitutive proteasomal degradation mediated by a multi‐subunit E3 ubiquitin ligase complex. Within this machinery, TRAF2 bridges cIAP1/2 to the NIK‐bound TRAF3, maintaining NIK at inactive trace levels [79, 89, 90, 91, 92]. Upon receptor ligation, downstream signalling triggers the degradation of this inhibitory TRAF/cIAP complex, liberating and stabilizing NIK. Consequently, the accumulated NIK—acting as the pivotal master regulator of this pathway—phosphorylates and activates IKKα, culminating in the essential proteolytic processing of the p100 precursor [57, 79, 81, 91, 92, 93].

Upon receptor engagement (e.g., via LTβ, CD40L or BAFF), the cIAP1/2‐TRAF2‐TRAF3 regulatory complex is recruited to the receptor, redirecting cIAP1/2‐mediated K48‐linked ubiquitination from NIK toward TRAF3 [94]. This targeted degradation of TRAF3 rescues NIK from constitutive proteolysis, allowing the stabilized kinase to phosphorylate IKKα, which subsequently orchestrates the phosphorylation‐dependent ubiquitination and processing of the p100 precursor into active p52 [79, 89, 90, 94]. The liberated RelB/p52 heterodimers translocate to the nucleus to drive specific transcriptional programs [89, 90], while NIK homeostasis is tightly fine‐tuned via a negative feedback loop driven by its downstream target, IKKα [95]. Beyond this core regulatory axis, NIK stability and function are restrained by several context‐specific negative regulators: TBK1‐mediated phosphorylation triggers NIK degradation in activated B cells [84], Monarch‐1 (NLRP12) drives NIK ubiquitination or stabilizes TRAF3 in myeloid compartments [96, 97], and TNAP directly suppresses NIK catalytic activity to halt p100 processing [98].

Beyond classical TNFR superfamily members, the non‐canonical NF‐κB cascade is engaged by diverse receptor systems to dictate lineage‐specific immune responses. For instance, OX40 (CD134) signalling drives effector and memory T cell expansion while antagonizing regulatory T cell (Treg) differentiation—an immunological shift implicated in countering the Treg accumulation characteristic of chronic viral hepatitis pathogenesis [78]. Similarly, non‐TNFRs such as the Macrophage Colony‐Stimulating Factor Receptor (M‐CSFR) utilize this pathway to govern macrophage proliferation and maturation [91, 92]. At the post‐transcriptional level, the central kinase IKKα is tightly restrained by specific microRNAs (miR‐223, miR‐15a and miR‐16), which act as negative rheostats to fine‐tune non‐canonical signalling during macrophage differentiation [99].

### 5.3 Alternative and Unconventional NF‐κB Pathways: Components, Activation, and Functional Consequences

**FIGURE 4:**
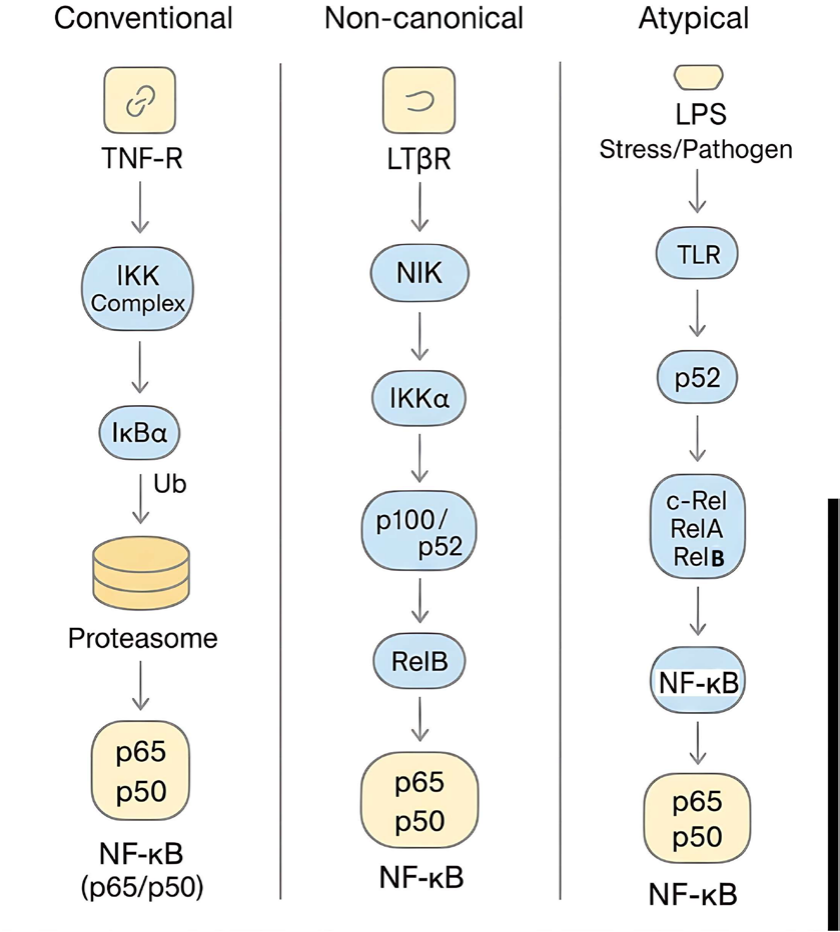
Schematic representation of the distinct NF‐κB signalling cascades: Canonical, Non‐canonical, and Atypical pathways. (Left) The canonical (conventional) pathway is classically initiated by ligand binding to cell‐surface receptors (e.g., TNF‐R), triggering the activation of the IKK holocomplex. This complex phosphorylates the inhibitory IκBα protein, marking it for ubiquitin‐proteasome‐mediated degradation, which subsequently liberates the classical p65/p50 heterodimer for nuclear translocation. (Middle) The non‐canonical pathway relies on specific receptor engagement (e.g., LTβR) to induce NF‐κB‐Inducing Kinase (NIK) accumulation. NIK subsequently activates IKKα, which orchestrates the proteolytic processing of the p100 precursor into p52. This processing facilitates the formation of active NF‐κB complexes (such as RelB/p52) to regulate specialized gene expression. (Right) The atypical (emergency) pathway is rapidly engaged in response to severe cellular stress, DNA damage, or pathogen‐associated molecular patterns (e.g., LPS via TLRs). This non‐traditional cascade bypasses conventional regulatory nodes, utilizing specialized intermediate complexes (NFC) and recruiting a diverse repertoire of NF‐κB subunits (including c‐Rel, RelA and p52) to mount a robust, stress‐responsive transcriptional defence.

In response to genotoxic stress, severe infections, or cellular damage, atypical NF‐κB signalling is engaged independently of the classical IKK holocomplex, frequently utilizing alternative kinases (e.g., NKK or unconventional IKKs). This versatile pathway drives the nuclear translocation of a broad spectrum of NF‐κB subunits (RelA, RelB, c‐Rel and p52) to orchestrate a robust DNA damage response (DDR). By coupling cell cycle checkpoint induction with DNA repair and pro‐survival transcriptional networks, unconventional NF‐κB activation prevents apoptosis and facilitates cellular recovery. However, this critical adaptive mechanism paradoxically acts as a primary driver of chemoresistance in neoplastic contexts, positioning these atypical pathways as highly strategic pharmacological targets in oncology [99, 100]. A comprehensive overview of these signalling cascades is illustrated in Figure 4.

### 5.4 Critical Summary: Exploiting Pathway Dichotomy for Precision Therapeutics

The structural and kinetic divergence among the canonical, non‐canonical and atypical NF‐κB cascades dictates a fundamental paradigm shift in targeted therapeutics. Historically, the undifferentiated blockade of the central IKK holocomplex has been severely hindered by collateral immunosuppression and paradoxical inflammatory flares, emphasizing the profound clinical peril of dismantling the rapid, IKKβ‐driven acute host defence mechanisms. Conversely, the non‐canonical axis—governed by the tightly regulated NIK‐IKKα signalling module—presents a highly specialized, slow‐burn transcriptional engine that predominantly drives chronic pathologies, particularly within hepatic, autoimmune, and lymphoid microenvironments. Furthermore, the atypical stress‐induced pathways act as crucial adaptive conduits for neoplastic chemoresistance. Therefore, the contemporary translational imperative lies in strategically exploiting this inherent mechanistic segregation. Future precision modalities must abandon blunt pan‐inhibition in favour of pathway‐selective antagonism—such as utilizing targeted protein degraders (PROTACs) against NIK, designing allosteric modulators for IKKα, or uncoupling atypical stress nodes. By meticulously decoupling pathological hyperactivation from essential physiological circuits, these sophisticated interventions can achieve robust disease modification while rigorously preserving basal innate immunity and cellular homeostasis.

## 6 Receptors Governing NF‐κB Regulation

### 6.1 Toll‐Like Receptors (TLRs)

Pattern recognition receptors (PRRs), encompassing Toll‐like Receptors (TLRs) and NOD‐like Receptors (NLRs), function as the vanguard of innate immunity by detecting exogenous Pathogen‐Associated Molecular Patterns (PAMPs) and endogenous Damage‐Associated Molecular Patterns (DAMPs) [101, 102]. Upon structural recognition of these motifs, PRRs ignite intracellular signalling cascades that drive cytokine and chemokine secretion to fortify host defence mechanisms [103]. Structurally, TLRs are characterized by a ligand‐binding Leucine‐Rich Repeat (LRR) domain, a transmembrane segment, and a cytosolic Toll/Interleukin‐1 Receptor (TIR) domain. Ligand engagement induces TLR dimerization, prompting TIR‐dependent recruitment of adaptor proteins and downstream kinases. Crucially, all TLR signalling trajectories converge on the activation of pivotal transcription factors—predominantly NF‐κB, alongside IRFs and AP‐1—to orchestrate a comprehensive inflammatory and antimicrobial transcriptional program [104, 105].

### 6.2 Toll‐Like Receptors (TLRs) and DAMP/PAMP Signalling

TLR signalling, predominantly via TLR4, functions synergistically with Nod‐like receptors (NLRs) to bridge innate and adaptive immunity [50]. While NLR activation triggers caspase‐1‐dependent secretion of IL‐1β and IL‐18 [103], TLRs orchestrate broader inflammatory cascades. Crucially, both exogenous PAMPs and host‐derived Damage‐Associated Molecular Patterns (DAMPs)—such as exposed mitochondrial components or injury‐induced mediators—converge on shared receptors like TLR4, thereby driving both infectious and sterile inflammation [106, 107, 108, 109]. Upon ligand recognition, TLRs homodimerize, inducing conformational shifts in their cytosolic TIR domains that facilitate the recruitment of the MyD88 adaptor protein [110]. The ensuing MyD88‐IRAK‐TRAF6 axis sequentially activates downstream kinases, notably TAK1 and MEKK1, which collectively stimulate the IKK complex [105, 111]. This cascade culminates in IκB degradation, licensing the nuclear translocation of key transcription factors, including NF‐κB, AP‐1 and IRF3, to execute comprehensive inflammatory transcriptional programs.

#### 6.2.1 Cell‐Specific Activation and Convergence of NF‐κB Pathways

Beyond typical immune cells, specific epithelial populations also mediate inflammation; for instance, keratinocytes utilize a CD14/TLR2/TLR4/MD2‐dependent MyD88 axis to process gut‐derived endotoxin signals [112, 113]. While TLRs are quintessential drivers of canonical NF‐κB activation, the Tumour Necrosis Factor Receptor Superfamily (TNFRSF) profoundly regulates both canonical and non‐canonical cascades under physiological stress. Diverse TNFRSF members (including BAFFR, CD40, LTβR, OX40, RANK and Fn14) and their cognate ligands assemble signalling hubs that recruit TRAF2, TRAF3 and cIAP1/2, initiating crucial ubiquitination events [57]. These TRAF adaptors bind the cytoplasmic domains of receptors such as CD30 and CD40—the latter being indispensable for B‐cell survival and differentiation [114, 115, 116] thereby functioning as platforms to recruit NF‐κB‐Inducing Kinase (NIK) [117]. Alternatively, TNFR1‐mediated signalling strictly necessitates the TRADD adaptor to engage the serine–threonine kinase RIP alongside TRAF proteins [118].

Ultimately, these diverse upstream signals converge on the IKK complex via distinct, cell‐type‐specific architectures. In innate immune cells, TLRs and TNFRSFs predominantly rely on MyD88/TRIF‐TRAF6 and TRAF2/5 axes, respectively [79, 119]. Conversely, in adaptive immunity, antigen receptor (BCR/TCR) engagement recruits PKCβ/PKCθ to assemble the CBM (Carma1/Bcl10/Malt1) signalosome. Upon IKK complex activation, the canonical pathway proceeds via IκBα degradation and p105 processing into the active p50 subunit. Concurrently, non‐canonical signalling hinges on the stabilization of NIK, which activates IKKα homodimers to mediate p100 processing, culminating in the nuclear translocation of active p52/RelB heterodimers [115, 119].

### 6.3 Tumour Necrosis Factor Receptors (TNFRs) and IL‐1 Receptors (IL‐1Rs)

Reflecting its pleiotropic nature, NF‐κB is robustly activated by a broad spectrum of stimuli, including environmental stressors and inflammatory cytokines (e.g., TNF, IL‐1 and IL‐18). Upon TNF stimulation, receptor engagement recruits the TRADD adaptor to mobilize specific TRAF family members; notably, TRAF2 orchestrates signalling downstream of both TNFR1 and TNFR2, while TRAF5 mediates responses from other TNFRSF members. Conversely, IL‐1 and IL‐18 signalling converge on TRAF6 [116]. Crucially, TNFR1 signalling bifurcates into pro‐survival NF‐κB/JNK pathways and a FADD/caspase‐8‐driven apoptotic cascade, highlighting a delicate cell‐fate dichotomy [120, 121].

In parallel, IL‐1 and IL‐18 propagate signals through a TRAF6/IRAK axis to activate NIK [122]. Mechanistically, IL‐1 signalling necessitates the assembly of a ternary complex comprising IL‐1RI and the accessory protein IL‐1RAcP. This complex recruits the PI3K p85 subunit, triggering a PI3K‐dependent cascade that culminates in robust NF‐κB activation and nuclear translocation. Furthermore, IL‐1R signalling functionally synergizes with T‐cell receptor (TCR) cascades, thereby amplifying and prolonging NF‐κB‐mediated transcriptional programs in adaptive immune responses.

NF‐κB‐Inducing Kinase (NIK), initially characterized as a TRAF2‐interacting MAP3K [122], physically couples with IKKα (formerly CHUK) to orchestrate downstream signalling [5]. While IKKα contributes to canonical cascades via IκBα phosphorylation (S32/S36) and subsequent proteasomal degradation [51, 123], the NIK/IKKα haα axis is primarily defined as the master regulator of the non‐canonical NF‐κB pathway. This alternate cascade is selectively engaged by specific TNFR superfamily members, such as LTβR, CD40, RANK, and BAFFR. Crucially, BAFFR dictates peripheral B‐cell survival and humoral homeostasis via robust NF‐κB2 (p100/p52) activation. Consequently, hyperactive BAFF signalling is a major pathogenic driver in autoimmune conditions like Systemic Lupus Erythematosus (SLE) and Sjögren's Syndrome, rendering the BAFF/NF‐κB signalling node a prime target for pharmacological inhibition.

### 6.4 B‐Cell Activating Factor Receptors (BAFFRs)

A specialized subset of the TNF receptor superfamily (TNFRSF)—comprising BAFFR, LTβR, CD40, RANK, CD27 and CD30—selectively orchestrates the non‐canonical NF‐κB2 (p1000/p52) cascade. The BAFF/BAFFR signalling axis, in particular, is indispensable for peripheral B‐cell maturation and humoral homeostasis. Pathologically, hyperactivated BAFF signalling drives autoimmune aetiologies such as Systemic Lupus Erythematosus (SLE) and Sjögren's syndrome. Consequently, this axis represents a critical therapeutic vulnerability, as its pathogenic expression can be pharmacologically antagonized by NF‐κB inhibitors.

### 6.5 Antigen Receptors

TCR and BCR engagement intrinsically couples with NF‐κB activation to govern adaptive immune homeostasis; conversely, signalling dysregulation precipitates chronic inflammation, autoimmunity, and lymphomagenesis. Rather than employing broad, systemic inhibition of shared downstream kinases (e.g., IKK), exploiting *receptor‐pathway specificity* emerges as a superior therapeutic paradigm. Precision targeting of receptor‐proximal nodes—specifically, adaptor proteins that dictate signal diversification (e.g., via TRAF ubiquitination) or distinct receptors like BAFFR—enables highly selective immunomodulation. This targeted strategy mitigates lymphocyte‐driven pathologies while circumventing the severe systemic toxicities and broad immunosuppression associated with global NF‐κB blockade.

### 6.6 Critical Summary: Spatiotemporal Targeting of Proximal Signalling Hubs

The architectural complexity of NF‐κB upstream signalling—spanning from broad TLR‐mediated innate sensing to the highly specialized BAFFR and antigen receptor cascades—presents a formidable paradigm of receptor pleiotropy and topological convergence. Historically, the phenomenon wherein diverse extracellular stimuli funnel into the central IKK complex has seduced researchers into targeting this ubiquitous downstream bottleneck. However, this generalized blockade has been repeatedly derailed in the clinic by severe systemic immunosuppression and intolerable physiological liabilities. Consequently, the contemporary translational imperative necessitates a strategic pivot toward receptor‐proximal precision targeting. By exploiting the distinct structural assemblies and scaffolding adaptors at the membrane‐proximal level—such as selectively antagonizing the CBM signalosome in aggressive lymphomas, selectively uncoupling TRAF‐dependent ubiquitination networks, or employing monoclonal antibodies against the BAFFR‐NIK axis in autoimmune aetiologies like systemic lupus erythematosus (SLE)—therapeutics can achieve unprecedented contextual specificity. Ultimately, shifting the pharmacological focus from the shared terminal kinases to these highly specific, stimulus‐driven proximal signalling hubs will effectively decouple pathological hyperinflammation from indispensable basal host defence, thereby radically widening the therapeutic index of next‐generation NF‐κB modulators.

## 7 The Role of Nutrition in Regulating NF‐κB

The intersection of nutrient‐sensing and immune pathways establishes a mechanistic link between metabolic dysregulation and overnutrition‐induced pathologies. A molecular hallmark of this crosstalk is the aberrant activation of the pro‐inflammatory IKKβ/NF‐κB axis within metabolic tissues, which drives a reciprocal disruption of metabolic homeostasis [124]. Unlike classical pathogen‐induced immunity, this condition—termed ‘metaflammation’—is defined by chronic, low‐grade, and sterile inflammation localized specifically to metabolic organs [125]. Dietary paradigms profoundly modulate this signalling hub; obesogenic ‘Western’ diets hyperactivate NF‐κB, whereas healthy regimens (e.g., Mediterranean diets) exert potent inhibitory effects. Consequently, leveraging precision nutrition to strategically titrate NF‐κB activity represents a highly translational therapeutic frontier for mitigating metaflammation‐driven chronic diseases.

Over‐nutrition fundamentally drives obesity‐associated metaflammation, a chronic, low‐grade systemic inflammatory state critically mediated by the NF‐κB pathway [126]. This pathogenic milieu is primarily initiated by the aberrant secretion of cytokines from hypertrophic adipose tissue [126, 127], synergized by microbiota‐derived Lipopolysaccharide (LPS) [128]. Mechanistically, circulating saturated fatty acids engage Toll‐like Receptors (TLRs), utilizing the MyD88 adaptor to hyperactivate intracellular inflammatory cascades, most notably IKKβ/NF‐κB and JNK [129]. While initiated via cell‐autonomous mechanisms, this signalling provokes the robust infiltration of immune cells (macrophages, dendritic cells and T cells) into key metabolic hubs—including the liver, skeletal muscle and vasculature—ultimately disrupting insulin receptor signalling and systemic homeostasis [129]. Crucially, the detrimental impact of IKKβ/NF‐κB‐driven metaflammation extends beyond peripheral tissues to the central nervous system. Hypothalamic metaflammation is now recognized as a primary catalyst for overnutrition‐induced metabolic syndrome, directly orchestrating the pathogenesis of obesity, insulin resistance, type 2 diabetes and associated hypertension [125, 129].

In Type 2 Diabetes, nutrient overload (hyperglycaemia and hyperlipidaemia) creates a self‐perpetuating inflammatory loop by constitutively activating the NF‐κB, MAPK and JAK–STAT signalling pathways [130, 131]. This metabolic stress triggers the release of pro‐inflammatory mediators from pancreatic islets and insulin‐sensitive tissues like adipose, which in turn recruits immune cells and escalates both local and systemic inflammation [132]. Within the pancreas, this glucolipotoxicity is particularly destructive; it drives elevated expression of TNFR5, leading to the pathological activation of NF‐κB and STAT1, culminating in islet inflammation and apoptotic loss of β‐cell mass [131]. Concurrently, peripheral insulin resistance and subsequent lipolysis elevate circulating Free Fatty Acids (FFAs), fostering severe lipotoxicity in non‐adipose tissues like the liver and muscle [133, 134]. In hepatocytes, these excess FFAs serve as substrates for ROS generation, activate PKCθ to exacerbate insulin receptor signalling defects, and induce lysosomal permeabilization with subsequent Cathepsin B release, thereby promoting cellular injury and apoptosis. This multi‐pronged hepatocyte damage directly correlates with the progression of fibrosis in Non‐Alcoholic Steatohepatitis (NASH) [134, 135].

Over‐nutritional dietary regimens, particularly those enriched with fructose and lipids, precipitate systemic metabolic dysfunction via dual pathological triggers: gut dysbiosis‐induced metabolic endotoxemia (LPS) and elevated circulating Free Fatty Acids (FFAs) [136]. These systemic insults universally engage Toll‐Like Receptors (e.g., TLR4) and TNFR1 across metabolic organs, driving the widespread accumulation and inflammatory polarization of immune cells that characterize obesity and Type 2 Diabetes [17, 137]. Within the liver, this translates into potent IKK/NF‐κB and JNK hyperactivation, which serves as a central driver for systemic insulin resistance, fibrosis, and the progression of Metabolic Dysfunction‐Associated Fatty Liver Disease (MAFLD) [127, 128, 136, 137, 138]. The obligate role of these upstream inflammatory sensors in carbohydrate‐ and lipid‐induced lipotoxicity is unequivocally demonstrated by findings that TLR4 genetic ablation or selective TNFR1 inhibition effectively abrogates dietary‐induced hepatic steatosis [104, 139, 140]. Furthermore, this over‐nutrition model establishes a highly refractory pathogenic loop; IKK‐mediated nuclear translocation of p65 (RELA) not only drives the transcription of classical pro‐inflammatory effectors (e.g., MCP‐1, VCAM‐1) but also actively upregulates ROS‐producing enzymes [17, 141]. This mutual reinforcement between intracellular oxidative stress and continuous NF‐κB signalling perpetuates the chronic, low‐grade pan‐tissue inflammation underlying central metabolic dysregulation [141].

Obesity‐induced overnutrition triggers profound endoplasmic reticulum (ER) stress across peripheral metabolic organs, critically impairing insulin signalling via JNK‐mediated serine phosphorylation of IRS‐1 [142, 143]. Central to this widespread metaflammation is the IKKβ/NF‐κB axis, which not only disrupts insulin action but also establishes a self‐amplifying pathological loop with upstream cytokine signalling networks [141, 143]. In the central nervous system, particularly the hypothalamus, high‐fat diet (HFD) feeding concurrently precipitates ER stress and IKKβ/NF‐κB hyperactivation. Notably, central IKKβ inhibition completely abrogates HFD‐ or chemically‐induced hypothalamic ER stress, underscoring the obligate role of IKKβ signalling in sustaining this cellular stress [144, 145]. Furthermore, the systemic metabolic consequences of hypothalamic IKKβ/NF‐κB activation exhibit striking neuronal specificity: signalling within Agouti‐Related Protein (AGRP) neurons preferentially drives energy dyshomeostasis and adiposity [144], whereas its activation in Pro‐opiomelanocortin (POMC) neurons dictates systemic hypertension and glucose intolerance [145, 146].

### 7.1 Critical Summary: Deconstructing the Metaflammatory Loop via Precision Pharmaconutrition

The conceptualization of overnutrition‐induced ‘metaflammation’ fundamentally repositions the IKKβ/NF‐κB cascade from a classical innate immune executor to a highly sensitive, systemic nutrient‐sensing rheostat. The pervasive and self‐amplifying nature of this metabolic‐inflammatory crosstalk—driven by the synergistic influx of circulating free fatty acids, microbiota‐derived endotoxemia, and unresolved endoplasmic reticulum (ER) stress—creates a formidable pathogenic loop spanning the hepatic, pancreatic, and central hypothalamic microenvironments. From a translational perspective, the profound cellular and neuronal specificity of this cascade (exemplified by the divergent systemic phenotypic outcomes of IKKβ hyperactivation in AGRP versus POMC neurons) exposes the severe mechanistic limitations of employing blunt, broad‐spectrum systemic anti‐inflammatory agents for metabolic syndrome. Therefore, the future therapeutic landscape dictates a paradigm shift toward precision ‘pharmaconutrition’ and spatiotemporally targeted modulators of the tissue‐specific TLR4‐IKKβ axis. By selectively uncoupling pathological nutrient‐sensing pathways from NF‐κB‐driven transcriptional networks, next‐generation interventions can elegantly dismantle the chronic metaflammatory loop, thereby rescuing systemic insulin sensitivity and metabolic homeostasis without compromising essential basal immunocompetence.

## 8 The Role of Oxidative Stress in the Regulation of NF‐κB

Hepatic oxidative stress, inextricably linked to lipid accumulation and insulin resistance, acts as a primary driver in the pathogenesis of liver disorders and metabolic syndrome [147]. Central to this oxidative milieu is NF‐κB, a highly redox‐sensitive transcription factor subject to bidirectional regulation by diverse oxidative stimuli [148, 149]. Reactive oxygen species (ROS) predominantly activate NF‐κB by inducing atypical phosphorylation of its inhibitor, IκBα [150, 151]. Notably, cellular stressors such as hypoxia/reoxygenation and pervanadate exposure trigger IκBα phosphorylation specifically at Tyrosine 42 (Y42). In stark contrast to canonical serine phosphorylation, this Y42 modification facilitates the direct dissociation of IκBα from NF‐κB without necessitating subsequent proteasomal degradation [150].

Intracellular mild oxidants, notably H_2_O_2_ generated downstream of diverse growth factors, cytokines, and GPCR agonists [152], engage the NF‐κB axis through multifaceted mechanisms. These include the atypical tyrosine phosphorylation of IκB and the concurrent serine phosphorylation of the p65 subunit [151]. Furthermore, oxidative stress indirectly amplifies IKK and Akt kinase activities by promoting the oxidative inactivation of key negative regulators, such as PTEN and IKK‐targeting phosphatases [148, 152]. However, this redox modulation exhibits significant structural and contextual complexity; for instance, H_2_O_2_ exposure or specific mutations in the NEMO regulatory subunit (e.g., C54/347A) can paradoxically blunt TNFα‐driven NF‐κB DNA binding and IKK activity [148]. Ultimately, sustained NF‐κB signalling fuels a pathological feed‐forward loop by transcriptionally upregulating cytotoxic and pro‐inflammatory mediators, thereby exacerbating oxidative stress and cellular dysfunction [141, 153]. Systemically, this vicious redox‐inflammatory cycle within metabolic and vascular compartments drives the pathogenesis of metabolic syndrome and its associated sequelae, including diabetes, atherosclerosis, stroke, cancer, and aging [154, 155].

The mechanistic nexus between obesity and oxidative stress is primarily driven by over‐nutrition, which overloads the mitochondrial electron transport chain (ETC), accelerating reactive oxygen species (ROS) generation alongside other sources like small GTPases [155, 156]. Interestingly, ROS‐induced NF‐κB activation diverges from the canonical IKK‐proteasome axis, relying instead on Src‐mediated tyrosine phosphorylation of IκBα [157]. This redox‐inflammatory signalling paradigm is central to obesity‐induced metabolic disturbances; elevated lipids and glucose trigger IKK, JNK and NF‐κB cascades specifically within the hypothalamus [144, 158]. Furthermore, this oxidative NF‐κB hyperactivation in neural networks fundamentally exacerbates metabolic syndrome‐associated neuropathologies, including stroke, neurodegeneration and brain aging [159, 160, 161, 162]. Counteracting these deleterious cascades, the histone deacetylase SIRT1 confers robust metabolic protection—mitigating atherosclerosis and aging sequelae—by potently attenuating ROS production and repressing NF‐κB transcriptional activity [163, 164].

### 8.1 Critical Summary: Decoding the Atypical Redox‐Inflammatory Nexus

The bidirectional, inextricably linked crosstalk between oxidative stress and the NF‐κB signalling apparatus fundamentally redefines this transcription factor—shifting its paradigm from a conventional immunological executor to a highly nuanced, context‐dependent redox sensor. The critical translational insight here lies in the atypical activation mechanics: unlike canonical pathogen‐driven cascades, redox insults bypass traditional IKK‐proteasomal bottlenecks, predominantly utilizing Src‐mediated tyrosine phosphorylation of IκBα (specifically at Y42) to achieve activation. This unique biochemical signature exposes the spatiotemporal duality of ROS; mild oxidative fluxes can paradoxically blunt canonical TNFα‐dependent activation via NEMO modifications, while sustained redox overload inevitably drives a pathological feed‐forward loop spanning peripheral metabolic tissues and the central hypothalamic networks. Consequently, deploying blunt, broad‐spectrum antioxidants or classical IKK inhibitors is therapeutically obsolete for resolving metaflammation. The contemporary pharmacological imperative must instead focus on uncoupling this vicious redox‐inflammatory cycle by selectively targeting non‐canonical, redox‐sensitive upstream nodes. Furthermore, exploiting epigenetic and metabolic safeguards—such as SIRT1 mimetics that dually repress ROS generation and NF‐κB transcriptional machinery—represents a highly promising, precision‐based therapeutic frontier for halting the progression of obesity‐driven cardiovascular and neurodegenerative sequelae.

## 9 Inflammation

Inflammation is an indispensable, multiphasic immune response deployed to neutralize deleterious stimuli—ranging from pathogens to sterile tissue damage—and subsequently restore physiological homeostasis [7, 165, 166, 167]. While acute, self‐limiting inflammation facilitates tissue repair, failure to resolve this initial cascade precipitates chronic inflammatory states. This unresolved chronicity is a primary driver of sustained pathologies, such as chronic liver diseases [168, 169, 170, 171]. Crucially, the progression and resolution of these inflammatory networks are intricately coupled with cellular metabolism. At the forefront of this immunometabolic interface are the energy sensors AMPK and Sirtuins. By continuously monitoring cellular AMP and NAD+ fluxes, these metabolic gauges orchestrate a pivotal shift from anabolic to catabolic states, inextricably linking inflammatory signalling to metabolic adaptations via epigenetic and transcriptional regulation [172].

The mutually antagonistic crosstalk between the evolutionarily conserved SIRT1 and NF‐κB pathways is central to immunometabolic homeostasis. This dynamic equilibrium ensures a rapid metabolic shift required to mount acute inflammatory responses against noxious stimuli, while simultaneously facilitating the subsequent resolution phase essential for restoring tissue integrity [171]. However, failure to resolve these cascades drives chronic inflammation, a quintessential pathogenic driver of multifaceted systemic disorders, including cardiovascular diseases, metabolic syndromes (e.g., Type 2 Diabetes), autoimmune conditions, and malignancies [173]. Notably, chronic infection and inflammation are implicated in approximately 15% of human oncogenesis [174]. Mechanistically, diverse sterile and infectious insults engage canonical pattern recognition and cytokine receptors (e.g., TLRs, IL‐1R, IL‐6R and TNFR). This receptor engagement precipitates the activation of classical intracellular signalling hubs—predominantly the NF‐κB, MAPK and JAK–STAT pathways—culminating in the robust release of pro‐inflammatory mediators such as IL‐1β, IL‐6, and TNF‐α [58, 64].

While acute inflammation serves as an essential, localized defence mechanism—driven by rapid microvascular permeability and chemokine‐directed leukocyte extravasation [175, 176, 177, 178]—its precise temporal and spatial regulation is paramount [179]. Mechanistically, this process follows a highly orchestrated cascade: initial detection of noxious stimuli by Pattern Recognition Receptors (PRRs) triggers convergent intracellular signalling networks, culminating in the release of inflammatory mediators and targeted cellular recruitment [58]. Crucially, a breakdown in the fine‐tuning of these pathways results in chronic, unresolved inflammation, representing a ubiquitous pathogenic driver across diverse systemic morbidities, including cardiometabolic, autoimmune and neoplastic disorders [180].

Averting the transition from acute to chronic inflammation necessitates an active, spatiotemporally regulated resolution phase governed by specialized pro‐resolving mediators [181]. This homeostatic restoration demands the dissipation of chemoattractant gradients to halt neutrophil infiltration [181], followed by a highly coordinated termination cascade: targeted apoptosis of exhausted neutrophils, bidirectional modulation of cytokine profiles, macrophage polarization toward a reparative phenotype, and subsequent tissue remodelling. Dysregulation within these finely tuned pathways fundamentally impairs resolution, invariably sustaining pathological chronicity [182, 183].

### 9.1 Critical Summary: Resolution Pharmacology and Immunometabolic Reprogramming

The contemporary understanding of inflammation transcends the classical paradigm of a simple host‐defence mechanism, redefining it as a highly dynamic, immunometabolic continuum. The critical inflection point dictating the trajectory toward either physiological restoration or pathological chronicity lies not merely in the initial receptor engagement, but in the active, spatiotemporally governed resolution phase. At the mechanistic core of this transition is the antagonistic interplay between the pro‐inflammatory NF‐κB transcriptional hub and cellular nutrient‐sensing rheostats, specifically the SIRT1 and AMPK axes. When this delicate immunometabolic equilibrium is perturbed, the failure to clear exhausted leukocytes and reprogram macrophage phenotypes precipitates a self‐perpetuating cycle of unresolved, sterile inflammation. Consequently, the translational frontier must fundamentally evolve beyond the conventional reliance on blunt anti‐inflammatory suppression, which invariably compromises essential, acute host defence. Instead, the future therapeutic landscape necessitates a strategic pivot toward ‘resolution pharmacology’. By leveraging specialized pro‐resolving mediators or metabolic reprogrammers to actively terminate NF‐κB‐driven cascades and restore the SIRT1‐mediated catabolic state, next‐generation interventions can elegantly dismantle the chronic inflammatory milieu underlying neoplastic and cardiometabolic sequelae without inducing deleterious systemic immunosuppression.

## 10 NF‐κB Inflammatory Markers

Inflammatory biomarkers have emerged as indispensable clinical tools for distinguishing pathological states and monitoring therapeutic efficacy. Persistent systemic inflammation fundamentally drives the aetiology and progression of diverse conditions, including cardiovascular diseases, endothelial dysfunction and severe infectious responses; thus, making these markers robust, independent prognostic indicators [184, 185]. Following pathological stimuli, innate immune cells (e.g., macrophages and adipocytes) release a cascade of pro‐inflammatory cytokines, prominently IL‐6, TNF‐α and IL‐1β. Profiling these specific molecular mediators provides critical diagnostic and prognostic utility, ultimately guiding targeted therapeutic interventions in chronic inflammatory disorders [186, 187].

While cytokines orchestrate essential leukocyte recruitment during immune responses [188], their unchecked hyperactivation precipitates severe systemic pathologies, culminating in extensive tissue damage and multi‐organ failure [189, 190]. Consequently, delineating the regulatory nodes of these pathways is imperative for advancing targeted anti‐inflammatory therapies [173]. Mechanistically, this inflammatory cascade is potently driven by Damage‐Associated Molecular Patterns (DAMPs) such as extracellular High‐Mobility Group Box 1 (HMGB1). HMGB1 directly engages Toll‐like Receptor 4 (TLR4), triggering MyD88‐dependent intracellular cascades that activate the NF‐κB and MAPK pathways, thereby driving the robust release of TNF‐α and IL‐1β [191, 192]. Furthermore, this profound inflammatory signalling is inextricably linked to oxidative stress. As the inflammatory milieu overwhelms endogenous antioxidant defenses, classical inflammatory biomarkers [64] are accompanied by a surge in Reactive Oxygen Species (ROS) and key indices of cellular oxidative damage, notably Malondialdehyde (MDA), 8‐hydroxy‐2‐deoxyguanosine (8‐OHdG), and isoprostanes [179, 193].

Activation of stress‐responsive transcription factors, including NF‐κB, AP‐1, p53, and STAT, drives the pathological overexpression of pro‐inflammatory mediators [194]. Consequently, oxidative stress byproducts serve as reliable surrogate biomarkers for monitoring systemic inflammation across diverse chronic pathologies [58]. The chronicity of this inflammatory cascade is particularly evident in recurrent liver injuries, where persistent NF‐κB hyperactivation in hepatocytes acts as the fundamental driver transitioning acute repair into chronic hepatic inflammation [195, 196]. Given its central pathogenic role, targeted NF‐κB inhibition is systematically evaluated using specific biomarker panels to determine therapeutic efficacy [42]. Clinically, this suppression strategy is highly efficacious in treating various hematological malignancies [9]. Furthermore, novel metabolic therapies, such as GLP‐1 receptor agonists, exert profound anti‐inflammatory effects primarily through NF‐κB attenuation. This inhibition effectively downregulates a broad spectrum of cellular (ROS, IL‐1β, TNF‐α, JNK1 and TLR2) and circulating (IL‐6, MCP‐1, MMP‐9 and SAA) inflammatory biomarkers [42].

### 10.1 Critical Summary: From Downstream Readouts to Upstream Transcriptional Targeting

While profiling circulating cytokines (IL‐6, TNF‐α and IL‐1β) and oxidative stress indices (MDA, 8‐OHdG) provides indispensable diagnostic and prognostic utility, it is critical to recognize these molecules primarily as downstream biological readouts rather than the core pathogenic drivers of chronic inflammation. The true mechanistic nexus orchestrating this multifaceted inflammatory milieu lies in the persistent activation of upstream intracellular hubs, predominantly the TLR4/MyD88/NF‐κB axis, which is continuously fuelled by endogenous DAMPs like HMGB1 and unmitigated ROS generation. Consequently, the paradigm of contemporary precision medicine must evolve from merely quantifying these surrogate markers toward pharmacologically dismantling their central transcriptional engine. The profound clinical efficacy of NF‐κB attenuation—demonstrated both in targeted oncological therapies for haematological malignancies and through the pleiotropic benefits of metabolic modulators such as GLP‐1 receptor agonists—underscores the superiority of this upstream intervention strategy. Moving forward, the strategic integration of robust biomarker panels should be explicitly repurposed to map pharmacodynamic target engagement, ensuring that novel immunometabolic interventions effectively and precisely extinguish the NF‐κB‐driven pathogenic core.

## 11 The Role of NF‐κB in Apoptosis and Disease Pathogenesis

### 11.1 The Dichotomous Function of NF‐κB in Apoptosis

The involvement of NF‐κB in programmed cell death is highly pleiotropic and strictly context‐dependent. Classically, it functions as a master cytoprotective regulator by driving the transcription of robust anti‐apoptotic networks, including IAP family members (cIAP, XIAP), Bcl‐2 homologues (Bcl‐XL, A1), cFLIP, TRAF1 and GADD45β [197]. This survival mechanism is indispensable in synovial cells and hepatocytes [198, 199]. In the hepatic niche, NF‐κB not only orchestrates tissue regeneration but also synergizes with JNK signalling following LPS or IL‐1β exposure to neutralize TNF‐induced cytotoxicity [24, 197, 200, 201, 202]. The criticality of this axis is highlighted in vivo, where RelA/p65 deletion precipitates embryonic lethality secondary to massive, unchecked TNF‐driven apoptosis—a phenotype entirely rescued in RelA/TNF double‐knockout models [203, 204]. Similarly, NF‐κB suppression via an IκBα super‐repressor exacerbates apoptotic vulnerability in double‐positive CD4/CD8 T‐cells [205].

Conversely, NF‐κB can paradoxically drive pro‐apoptotic cascades in specific cellular contexts. It actively collaborates with the AP‐1 transcription factor to induce FasL expression [206, 207] and exacerbates cell death in neuronal populations and T cell hybridomas. Uniquely, overexpression of the c‐Rel subunit is sufficient to trigger apoptosis in avian embryos and bone marrow models [208, 209]. Pathologically, the potent pro‐survival functions of NF‐κB are frequently hijacked; for instance, while PRR‐mediated activation in keratinocytes mounts vital immune responses impacting hepatic inflammation [210], its chronic hyperactivation in the liver shields malignant hepatocytes from apoptotic elimination, thereby driving oncogenesis [211]. Consequently, therapeutic ablation of the NF‐κB pathway, particularly via IKKβ inhibition, effectively dismantles the anti‐apoptotic shield (Bcl2, IAP and cFLIP) of cancer cells, significantly sensitizing them to chemotherapeutic agents [9].

### 11.2 NF‐κB Dysregulation as a Driver of Chronic Systemic Pathologies

The NF‐κB signalling axis serves as the master orchestrator of immune‐inflammatory homeostasis, yet its chronic aberration underpins the pathophysiology of diverse systemic disorders [91]. A pivotal hallmark of aging is ‘Inflamm‐aging’, characterized by immunosenescence and a compromised redox‐defence capacity. This state triggers a persistent, low‐grade inflammatory cascade driven by NF‐κB‐mediated activation of pro‐inflammatory genes [212, 213]. Upon oxidative or cytokine‐mediated stimulation (e.g., TNF‐α, IL‐1β), NF‐κB governs a vast secretome, including chemokines (RANTES, CINC), adhesion molecules, and enzymes like COX‐2, which collectively facilitate the recruitment and maturation of macrophages and neutrophils [214, 215, 216, 217]. In conditions such as gout, obesity and asthma, the M1/M2 macrophage polarization and subsequent IL‐1β surges are primarily dictated by this pathway [218, 219, 220].

The clinical footprint of NF‐κB hyperactivation extends to cardiovascular and metabolic syndromes. Dysregulated NF‐κB flux, often intersecting with MAPK and JAK/STAT cascades, is a critical risk factor for atherosclerosis, hypertension, and heart failure [79, 221, 222, 223, 224]. Furthermore, site‐specific p65 phosphorylation by Protein Kinase CK2 has been implicated in the pathogenesis of glomerulonephritis and gastritis, particularly following *H. pylori* infection [223, 224, 225]. In the context of adaptive immunity, persistent NF‐κB signalling drives inappropriate T‐cell activation, fostering autoimmune phenotypes [91]. Crucially, NF‐κB bridges inflammation and oncogenesis by establishing a tumour‐promoting microenvironment that precedes malignancy, thereby facilitating cell survival and immune evasion [9, 20]. Consequently, maintaining the delicate equilibrium between canonical and non‐canonical signalling is fundamental to preventing the transition from acute physiological responses to chronic pathological states [226].

### 11.3 NF‐κB as a Mechanistic Link in Insulin Resistance

In the context of obesity, NF‐κB acts as a central mediator of ‘metaflammation’—a chronic, low‐grade inflammatory state that impairs metabolic homeostasis. Activation of the NF‐κB axis drives ROS generation, precipitating oxidative injury that culminates in β‐cell dysfunction and disrupted insulin signalling. Mechanistically, elevated free fatty acids (FFAs) serve as potent triggers for both NF‐κB and JNK1 pathways, establishing a deleterious pro‐inflammatory milieu that reinforces systemic insulin resistance [16, 227, 228, 229].

### 11.4 NF‐κB as a Central Driver and Therapeutic Target in Oncogenesis

Chronic inflammation, orchestrated by the NF‐κB signalling axis, is a well‐established catalyst for tumorigenesis across a broad spectrum of malignancies [9, 20, 230, 231]. NF‐κB family members critically drive cancer progression by modulating a complex transcriptional program essential for cell survival, proliferation, angiogenesis, metastasis and chemoresistance. Notably, oncogenic stimuli such as *Ras*, *Rac* and *Bcr‐Abl* often amplify NF‐κB signalling, further cementing its role in malignant transformation [9]. The aberrant activation of NF‐κB within both tumour cells and the tumour microenvironment (TME)—including stromal and endothelial compartments—is pivotal for establishing a pro‐survival milieu. By upregulating anti‐apoptotic effectors, NF‐κB acts as a rate‐limiting safeguard against programmed cell death in numerous solid tumours (e.g., melanoma, breast and pancreatic cancers) and haematological malignancies (e.g., Hodgkin's lymphoma and multiple myeloma) [9, 232, 233, 234, 235, 236].

Beyond canonical signalling, IKKβ facilitates tumorigenesis through a dual‐track mechanism: activating NF‐κB and simultaneously inhibiting the tumour suppressor *FOXO3a* via phosphorylation‐dependent proteasomal degradation [237]. This mechanistic synergy underscores the potential of NF‐κB as a high‐value therapeutic node. Targeted modulation using dietary bioactive compounds—such as polyphenols and alkaloids (e.g., curcumin and capsaicin)—has shown promise in suppressing NF‐κB via inhibition of IKK activity and IκBα degradation [72, 238, 239, 240]. However, achieving clinical efficacy necessitates a shift from broad inhibition to the design of selective pharmacological inhibitors targeting discrete pathway components. Such precision strategies, grounded in validated dose‐correlation studies, aim to maximize anti‐tumour potency while mitigating systemic toxicities [9, 237].

### 11.5 The Role of NF‐κB in Hepatic Pathogenesis

NF‐κB acts as a master orchestrator of hepatic homeostasis and disease, driving the pathogenesis of non‐alcoholic fatty liver disease (NAFLD), fibrogenesis and hepatocellular carcinoma (HCC). In hepatocytes, the IKK/NF‐κB axis governs the transition from simple steatosis to NASH by concurrently regulating lipogenesis, cholesterol synthesis, and inflammatory activation [61, 127, 241, 242, 243]. Notably, chronic NF‐κB activity intrinsically exacerbates lipid accumulation and steatosis independent of lobular inflammation, while systemically correlating with elevated pro‐inflammatory cytokines (e.g., TNF, IL‐6 and IL‐1β) in obese phenotypes [17, 244, 245].

Beyond metabolic dysfunction, TLR4‐mediated NF‐κB signalling in response to PAMPs and DAMPs drives innate immune activation across endotoxemia, alcohol‐related liver disease (ALD) and ischaemia/reperfusion (I/R) injury [242, 246, 247]. This signalling cascade critically promotes fibrogenesis by activating hepatic stellate cells (HSCs), downregulating the TGF‐β pseudoreceptor BAMBI, and upregulating anti‐apoptotic effectors like Bcl‐2 to ensure HSC survival [248, 249]. Interestingly, NF‐κB exerts a dichotomous role in hepatic injury. While its chronic activation underpins non‐viral HCC etiologies associated with metabolic syndrome, hepatocyte‐specific NF‐κB deletion precipitates spontaneous liver damage, ROS accumulation, and compensatory proliferation, paradoxically facilitating tumorigenesis [9, 250, 251, 252, 253]. Furthermore, NF‐κB activation drives drug‐induced liver injury (DILI) through OX40‐dependent mechanisms [254, 255].

Recent evidence heavily implicates the non‐canonical pathway, specifically NF‐κB inducing kinase (NIK), as a critical driver of NAFLD progression and metabolic abnormalities [254, 256, 257]. NIK also mediates antiviral defense by inducing APOBEC3B‐dependent degradation of HBV cccDNA via LTBR signalling, although the HBV polymerase actively antagonizes this non‐canonical immune response [258, 259]. The overall pathway activity is further amplified by Casein Kinase 2 (CK2), which phosphorylates multiple upstream and downstream nodes including IKK, IκB and the p65 subunit [260, 261, 262].

Therapeutically, modulating this intricate network offers immense clinical potential. Emerging strategies include enhancing hepatic insulin sensitivity via High‐Fat Diet (HFD)‐induced, SIRT1‐mediated p65 acetylation, utilizing plant‐derived flavonoids like apigenin, or deploying small‐molecule selective NIK inhibitors (e.g., XT2, B022) to effectively ameliorate drug‐related toxicity, systemic inflammation, and metabolic hepatic injuries [254, 263, 264].

### 11.6 The Role of NF‐κB in Autoimmune Diseases

The non‐canonical NF‐κB pathway, orchestrated by the NIK‐IKKα axis, is fundamentally required for establishing central immune tolerance [78]. Epithelial‐specific ablation of NIK or IKKα in medullary thymic epithelial cells (mTECs) arrests their differentiation, precipitating a profound breakdown in central T‐cell tolerance. This immunological defect directly drives the aetiology of fatal, acute T cell‐mediated autoimmune hepatitis, characterized by severe liver fibrosis and premature mortality in murine models [78, 265].

Beyond thymic regulation, systemic NF‐κB and B‐cell hyperactivation across various autoimmune and inflammatory landscapes are clinically reflected by elevated circulating Free Light Chains (FLCs), which emerge as surplus byproducts of antibody synthesis [8, 266]. Consequently, polyclonal circulating FLCs serve as independent prognostic biomarkers for overall survival (OS) and mortality risk [267]. Notably, in rheumatic diseases, this hyperactivation manifests as significantly upregulated levels of Kappa FLC (FLCκ) and a markedly elevated FLCκ/λ ratio compared to healthy cohorts [268].

Beyond their role in immune tolerance, Free Light Chains (FLCs) have emerged as potent diagnostic and therapeutic indicators, directly reflecting disease activity in inflammatory and autoimmune contexts [269]. Central to this pathology is the chronic overactivation of NF‐κB, which drives a wide clinical spectrum from uveitis to Rheumatoid Arthritis (RA). In RA, NF‐κB (specifically the p50 and p65 subunits) orchestrates a pro‐inflammatory milieu within synovial fibroblasts, triggering the sustained release of IL‐1, IL‐6 and TNF‐α. This signalling cascade is a primary driver of synovial inflammation and subsequent cartilage and bone erosion [14, 30].

Furthermore, the impact of NF‐κB mediated inflammation extends to cardiac pathologies, notably Atrial Fibrillation (AF). Emerging evidence identifies immune‐inflammatory mechanisms as critical factors in the initiation and progression of AF, positioning these pathways as key research frontiers [270, 271]. Given these complexities, bioactive nutraceuticals like curcumin have gained attention; clinical trials indicate that curcumin can significantly ameliorate the clinical symptoms of RA and uveitis by modulating these inflammatory axes, offering a promising supplementary therapeutic strategy [272].

### 11.7 The Role of NF‐κB in Biliary and Pancreatic Diseases

In biliary pathologies, the non‐canonical NF‐κB subunit, RelB, serves as a critical driver of the ductular response and the activation of hepatic progenitor (oval) cells, ultimately exacerbating biliary fibrosis [273]. Furthermore, the BAFF‐mediated activation of non‐canonical NF‐κB signalling in B cells represents a pathogenetic hallmark in the progression of Primary Biliary Cirrhosis (PBC) [274]. The therapeutic potential of targeting this axis is underscored by evidence that NF‐κB or proteasome inhibitors significantly mitigate hepatic fibrosis in bile duct ligation (BDL) models [275].

Parallel to biliary disorders, NF‐κB acts as a central node in the inflammatory orchestration of pancreatitis, regardless of the aetiology (e.g., ductal obstruction, genetic mutations or ethanol consumption). Its synergistic crosstalk with the MAPK and JAK–STAT pathways is essential for the systemic activation of inflammatory cells, driving the clinical progression of pancreatic inflammation [276].

### 11.8 The Role of NF‐κB in Cardiovascular Diseases

The transcription factor NF‐κB functions as a master regulator in the pathogenesis of various cardiovascular diseases (CVDs). It acts as a central hub that transduces environmental risk factors—such as smoking and chronic viral infections (e.g., HIV, HBV)—as well as oxidative stress into robust pro‐inflammatory cascades [20, 42, 277, 278].

In the context of atherogenesis, NF‐κB activation within lesional macrophages precipitates a critical feed‐forward inflammatory loop by upregulating pivotal mediators, including TNF‐α, IL‐1β, IL‐6, MCP‐1 and ICAM‐1. Genetic and pharmacological evidence corroborates that targeted inhibition of this axis effectively blunts atherosclerotic plaque progression in hyperlipidemic models [42, 279].

Beyond vascular lesions, localized NF‐κB hyperactivation orchestrates myocardial inflammation. It is a primary driver of encephalomyocarditis virus (EMCV)‐induced myocarditis and promotes profound lymphomonocyte infiltration in the atria, establishing the inflammatory arrhythmogenic substrate for atrial fibrillation (AF) [270, 271, 280]. Furthermore, systemic cardiovascular risk is profoundly amplified by metabolic crosstalk; hepatic NF‐κB signalling drives de novo lipogenesis and cholesterol synthesis, inextricably linking metabolic‐associated fatty liver disease (MAFLD) to an exacerbated CVD risk profile [67, 72].

### 11.9 The Role of NF‐κB in the Aging Process

Aging‐associated cardiovascular decline and metabolic dysregulation are mechanistically tethered to endothelial NF‐κB hyperactivation. Targeted suppression of the endothelial NF‐κB signalling cascade (e.g., via endothelium‐specific overexpression of IκBα) not only extends lifespan but also actively preserves systemic metabolic homeostasis. This targeted inhibition effectively mitigates obesity‐induced insulin resistance in sensitive peripheral tissues and blunts lesional macrophage infiltration [281].

Furthermore, during senescence, unbridled NF‐κB activation drives structural and functional deterioration, culminating in impaired endothelium‐dependent vasodilation, systemic ‘inflammaging’ and progressive myocardial fibrosis [282, 283]. Consequently, targeted pharmacological modulation of the NF‐κB axis emerges as a compelling geroprotective strategy to attenuate and potentially reverse age‐related cardiovascular and metabolic pathologies [42].

### 11.10 The Role of NF‐κB in Metabolic Diseases

The metabolic influence of NF‐κB is tightly regulated by SIRT1 through the direct deacetylation of the p65 subunit, a modification that dictates its nuclear exclusion and subsequent transcriptional silencing. Crucially, a mutually antagonistic interplay exists between SIRT1 and NF‐κB within the hypothalamus, the master regulatory center of systemic energy homeostasis. Dysregulation of this central hypothalamic axis disrupts the neuro‐metabolic circuitry, precipitating a cascading failure in peripheral energy balance and establishing the SIRT1/NF‐κB crosstalk as a foundational pathogenic nexus in metabolic diseases [171].

### 11.11 The Role of NF‐κB in Inflammatory Bowel Disease (IBD)

The pathogenesis of inflammatory bowel diseases (IBD), encompassing Crohn's disease (CD) and ulcerative colitis (UC), is fundamentally driven by the aberrant hyperactivation of the NF‐κB signalling cascade. Clinical biopsy analyses and immunohistochemical data consistently reveal robust nuclear translocation of the p65 subunit within the intestinal epithelium and lamina propria macrophages during active disease states [284, 285, 286, 287]. This localized hyperactivation orchestrates profound immune dysregulation, predominantly by upregulating macrophage‐derived Th1 cytokines, such as IL‐12, thereby perpetuating chronic mucosal damage and structural barrier dysfunction [5, 288].

Given its persistent activation in the inflamed gut, p65 has emerged as a quintessential therapeutic target. Experimental blockade using p65‐specific antisense oligonucleotides effectively abrogates pro‐inflammatory cytokine secretion and ICAM‐1 cell adhesion molecule expression, successfully inducing robust disease remission in murine colitis models. Furthermore, the clinical efficacy of established conventional treatments, notably glucocorticoids, is largely attributed to their potent suppression of this transcription factor [284, 289, 290]. Consequently, the targeted molecular attenuation of NF‐κB holds immense translational promise for developing next‐generation IBD therapeutics, offering enhanced clinical specificity with substantially minimized systemic toxicity [5, 291].

### 11.12 The Role of NF‐κB in Kidney Disease and IVDD

Pathological activation of NF‐κB serves as a fundamental convergence point in the progression of both renal injury and intervertebral disc degeneration (IVDD). In the renal microenvironment, a diverse array of pathological stimuli—encompassing cytokines, DAMPs, PAMPs and metabolic stressors like advanced glycation end‐products (AGEs)—engage pattern recognition receptors (e.g., TLRs, NLRs) to trigger robust NF‐κB and MAPK signalling cascades, thereby driving renal tissue damage [292].

Similarly, in the pathogenesis of IVDD, IL‐1β acts as a primary catabolic driver that disrupts extracellular matrix (ECM) homeostasis and precipitates cellular apoptosis [293, 294, 295]. Mechanistically, NF‐κB functions as the obligate downstream effector of this IL‐1β pathway, specifically orchestrating the deleterious upregulation of matrix metalloproteinases (MMPs) [296, 297].

Crucially, this destructive inflammatory axis is tightly counter‐regulated by the epigenetic modulator SIRT1. Disease progression is exacerbated when IL‐1β exposure downregulates SIRT1, simultaneously promoting TLR2 expression alongside the hyperacetylation and nuclear translocation of the NF‐κB p65 subunit. Conversely, targeted overexpression of SIRT1 effectively neutralizes this cascade; it preserves ECM integrity by directly deacetylating p65, which forcefully silences NF‐κB transcriptional activity and subsequently blunts IL‐1β mediated MMP and TLR2 upregulation [297].

**FIGURE 5:**
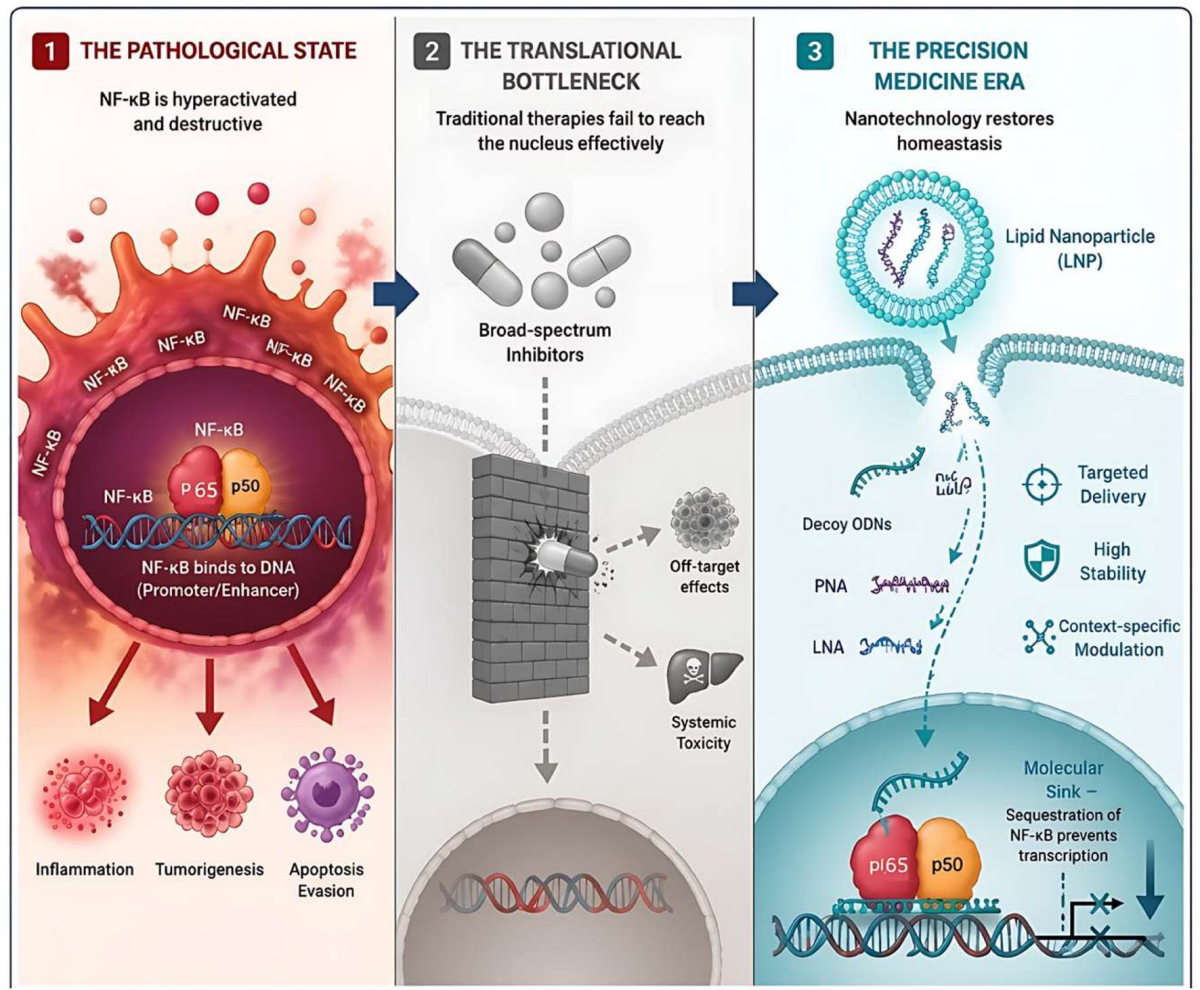
The therapeutic evolution of NF‐κB targeting: From translational bottlenecks to precision nanomedicine. (1) The Pathological State: constitutive hyperactivation of the NF‐κB heterodimer (e.g., p65/p50) drives the transcription of target genes, resulting in robust inflammatory responses, tumorigenesis, and apoptosis evasion. (2) The Translational Bottleneck: conventional broad‐spectrum pharmacological inhibitors often fail to achieve optimal nuclear efficacy, leading to severe pharmacokinetic liabilities, including off‐target effects and dose‐limiting systemic toxicity (hepatic/systemic clearance). (3) The Precision Medicine Era: next‐generation targeted delivery systems, notably Lipid Nanoparticles (LNPs), facilitate the intracellular release of advanced nucleic acid therapeutics (Decoy ODNs, PNAs and LNAs). These engineered molecules act as a ‘molecular sink’, competitively sequestering the NF‐κB complex, preventing its binding to endogenous promoter/enhancer regions, and effectively restoring cellular homeostasis without systemic toxicity.

The paradigm shift in overcoming the translational bottlenecks of NF‐κB modulation, highlighting the critical role of next‐generation delivery systems such as LNPs, is conceptually illustrated in Figure 5.

#### 11.12.1 Critical Summary: Resolving the Pharmacological Conundrum of Apoptosis

While the canonical paradigm positions NF‐κB as an obligate survival factor, its context‐dependent pro‐apoptotic capacity reveals a profound pharmacological conundrum. The therapeutic exploitation of this axis in oncology cannot rely on blunt pan‐inhibition, which risks catastrophic collateral damage to vital hepatic and immune homeostasis. Instead, next‐generation interventions must pivot toward ‘context‐aware’ precision targeting—disentangling the pathogenic anti‐apoptotic shield in malignant niches from physiological survival networks. This necessitates mapping the precise spatiotemporal dynamics and co‐regulatory transcription factors (e.g., AP‐1) that dictate this dichotomous switch, ultimately enabling synthetic lethal strategies that selectively unmask cancer cells to apoptosis without precipitating systemic toxicity.

#### 11.12.2 Mastering the Rheostat of Systemic ‘Metaflammation’

The ubiquitous involvement of NF‐κB across disparate systemic pathologies—from immunosenescence and atherosclerosis to autoimmunity and oncogenesis—cements its status as the master rheostat of systemic inflammation. However, this extreme pleiotropy represents a double‐edged sword for clinical translation. The pervasive failure to effectively manage chronic inflammatory syndromes often stems from treating downstream phenotypic endpoints rather than their shared transcriptional root. A paradigm shift is imperative: transitioning from generic immunosuppression to the precise spatiotemporal modulation of NF‐κB flux. By selectively targeting tissue‐specific upstream kinases or post‐translational modulators (e.g., CK2), future therapeutics can successfully dissect the physiological acute‐phase host defence from pathological chronic hyperactivation, thereby resolving ‘inflammaging’ and autoimmunity at their inception.

#### 11.12.3 Reframing Insulin Resistance as an Immunometabolic Disorder

The mechanistic convergence of the NF‐κB and JNK1 signalling cascades in response to lipotoxic stress definitively reframes insulin resistance from a classical endocrinological defect to a core immunometabolic disorder. This lipo‐inflammatory feed‐forward loop, persistently driven by elevated FFAs and ROS, highlights the inherent limitations of conventional metabolic therapies that fail to extinguish the underlying inflammatory epicentre. To effectively reverse obesity‐induced insulin resistance and preserve β‐cell integrity, pharmacological strategies must seamlessly integrate immunomodulation with metabolic correction. Uncoupling the cellular metabolic sensors from their inflammatory effectors—specifically by blunting the FFA‐NF‐κB axis without disrupting basal energy sensing—represents the critical next frontier in the precision management of metabolic syndrome.

## 12 Novel Therapeutic Approaches

### 12.1 Pharmacological Inhibitors of NF‐κB

As established, NF‐κB operates as a master transcriptional regulator of immune homeostasis, the dysregulation of which is intrinsically linked to the pathogenesis of chronic inflammation, autoimmunity and oncogenesis [298]. While this central role positions the NF‐κB signalling axis as a highly compelling therapeutic target, the clinical translation of classical pharmacological inhibitors remains severely hindered by significant bottlenecks. Chief among these challenges is achieving selective and targeted delivery. Current conventional approaches often suffer from sub‐optimal efficacy and profound pharmacokinetic liabilities, including off‐target systemic toxicities resulting from indiscriminate inhibition, the inherent instability of biological macromolecules, rapid in vivo degradation of nucleic acids, and non‐selective biodistribution.

While conventional therapeutics, notably glucocorticoids, effectively suppress NF‐κB‐driven inflammation, their chronic application is severely hindered by a spectrum of debilitating off‐target morbidities, including osteoporosis, metabolic dysregulation, and heightened susceptibility to opportunistic infections [299]. This clinical imperative has catalyzed a paradigm shift toward next‐generation precision interventions that balance robust anti‐inflammatory efficacy with superior safety profiles.

Among these emerging modalities—which span low molecular weight compounds and biologic agents—Oligonucleotide Therapeutics (ONTs) have garnered prominent attention. As a sophisticated class of nucleic acid‐derived tools, ONTs encompass antisense oligonucleotides (ASOs), small interfering RNAs (siRNAs), microRNAs (miRNAs), aptamers and decoy oligodeoxynucleotides. Collectively, these agents offer a highly specific, transcriptomic‐level approach to attenuate aberrant signalling pathways, successfully bypassing the profound limitations of classical pharmacotherapy [300].

Oligonucleotide therapeutics boast intrinsic advantages over traditional biologics, including superior stability, scalability, and cost‐efficiency. Leveraging chemical modifications and advanced delivery platforms, such as polymeric nanocarriers, substantially prolongs their half‐life and shields them from enzymatic degradation. Nonetheless, their translational success is strictly dictated by targeted biodistribution. For instance, in hepatic fibrogenesis, activated hepatic stellate cells (HSCs) represent critical therapeutic loci; however, the scarcity of unique HSC surface markers necessitates the design of sophisticated, targeted nanocarriers to ensure specific uptake and preclude off‐target toxicity in healthy parenchyma [301].

Concurrently, natural phytochemicals—particularly polyphenolic flavonoids—have gained significant traction as high‐efficacy, low‐toxicity candidates for both oncological and inflammatory management. Their therapeutic potency stems largely from their ability to dismantle NF‐κB‐driven survival pathways and quench oxidative stress [302]. Notably, phloretin, a plant‐derived dihydrochalcone, exerts robust anti‐inflammatory efficacy by directly abrogating NF‐κB activation, thereby suppressing the downstream transcription of pro‐inflammatory cytokines and orchestrating immune homeostasis [303].

First‐generation cytokine immunotherapies, while capable of eliciting durable anti‐tumour responses, are fundamentally hindered by suboptimal pharmacokinetics and severe systemic toxicity, necessitating advanced strategies to widen their therapeutic index [304]. At the molecular level, the IκBα super‐repressor serves as a highly selective tool for targeted NF‐κB inhibition. As a dominant‐negative mutant resistant to inducible phosphorylation and subsequent ubiquitination, it ensures sustained NF‐κB blockade [305, 306]. Crucially, unlike IκBα phosphopeptide inhibitors, this super‐repressor circumvents NF‐κB‐independent β‐TrCP interactions, offering superior specificity [9].

Furthermore, the upstream activation of IKKβ is highly context‐dependent, intricately modulated by chromatin architecture, cellular lineage, and inflammatory microenvironmental cues [9]. Beyond NF‐κB activation, IKKβ exerts pleiotropic effects, notably by neutralizing tumour suppressors such as FOXO3a via phosphorylation‐driven nuclear exclusion and degradation [237, 307]. Consequently, rational drug design necessitates rigorous early‐stage profiling of multi‐target pleiotropy and off‐target activities—such as inadvertent alterations in IL‐1 signaling—to minimize adverse events and optimize NF‐κB‐targeted therapeutic regimens [9].

The pharmacological armamentarium for NF‐κB blockade encompasses a broad spectrum of modalities. Upstream kinases can be inactivated via alkylating agents and antioxidants, whereas specific transcriptional arrest of the p65p65p65 subunit is achievable through antisense DNA and glucocorticoids [117]. Gene therapy employing adeno‐associated viral (AAV) and adenoviral vectors offers robust delivery platforms for NF‐κB silencing; however, their clinical translation is significantly bottlenecked by vector immunogenicity. Pre‐existing neutralizing antibodies against viral capsids can severely diminish therapeutic efficacy [287]. Paradoxically, the robust immunostimulation induced by adenoviral vectors acts as a double‐edged sword: while it confers advantageous anti‐tumour adjuvant effects in oncology, it simultaneously precipitates rapid vector clearance and systemic toxicity [304].

A critical evaluation of current anti‐inflammatory therapeutics reveals a complex balance between efficacy and safety. Conventional agents, such as glucocorticoids (e.g., Dexamethasone) and small‐molecule inhibitors (e.g., Bortezomib, BAY 11‐7082), provide rapid and potent NF‐κB suppression; yet, their long‐term utility is heavily compromised by severe systemic sequelae, off‐target toxicity, and dose‐limiting immunosuppression. Conversely, biologic therapies (e.g., anti‐TNF‐α and anti‐IL‐1β monoclonal antibodies) offer precise targeting and prolonged half‐lives, but are encumbered by prohibitive costs, parenteral administration constraints, and paradoxical immunogenicity. Advanced nucleic acid platforms, including synthetic miRNAs, antagomirs and decoy oligodeoxynucleotides (ODNs), promise highly specific, transcriptional‐level regulation. Nevertheless, their success remains strictly dependent on overcoming nuclease‐mediated degradation and intracellular delivery hurdles. Ultimately, natural phytochemicals (e.g., flavonoids, curcumin and phloretin) emerge as highly favourable candidates with multimodal anti‐inflammatory and cytoprotective profiles, though their broader clinical application necessitates novel formulations to resolve inherent bioavailability bottlenecks and rapid metabolic clearance.

#### 12.1.1 NF‐κB Decoy Oligodeoxynucleotides (ODNs)

Decoy oligodeoxynucleotides (ODNs) represent a highly targeted, sequence‐specific therapeutic modality designed to directly antagonize the transcriptional activity of NF‐κB, a master pleiotropic regulator of immune dynamics, cellular proliferation and apoptosis [308]. Mechanistically, these synthetic double‐stranded DNA constructs harbour consensus κB binding motifs that act as molecular sinks. By competitively sequestering native NF‐κB dimers within the intracellular compartment, decoy ODNs effectively abrogate their interaction with endogenous promoter *cis*‐elements. This targeted transcriptional blockade robustly silences downstream pro‐inflammatory cascades, elegantly bypassing the complex network of upstream signalling pathways. For instance, this strategy potently abolishes TNF‐α‐mediated expression of IL‐6 and IL‐8 in vitro.

Unlike conventional small‐molecule inhibitors that target upstream kinases and often exhibit broad off‐target toxicities, the decoy approach provides superior molecular precision by exclusively intercepting the terminal DNA‐binding event—an absolute prerequisite for gene transcription. Nevertheless, while this strategy ensures profound inhibition of the inflammatory master regulator, its clinical translation is currently challenged by the critical need for tissue‐selective delivery. Achieving targeted localization is imperative to circumvent the deleterious suppression of essential, basal NF‐κB physiological functions in healthy tissues.

Despite the rapidly expanding landscape of advanced RNA‐based therapeutics (e.g., siRNAs, mRNAs), NF‐κB‐targeting DNA decoys maintain substantial clinical relevance across a broad pathological spectrum, encompassing oncology, rheumatology, and cardiovascular medicine [309]. However, the translational success of these oligonucleotides is fundamentally hindered by the inherent pharmacokinetic vulnerability of their wild‐type phosphodiester backbone, which is subject to rapid intra‐ and extra‐cellular nucleolytic degradation [310, 311, 312].

To circumvent these profound metabolic barriers, advanced architectural modifications and delivery platforms—such as hairpin conformations, peptide nucleic acids (PNAs) and decoy sensors—have been engineered. The most prevalent structural adaptation is the phosphorothioate (PS) modification, involving the substitution of a non‐bridging oxygen within the phosphate linkage with sulfur. While PS‐ODNs confer remarkable nuclease resistance and enhanced cellular uptake, they inevitably introduce significant biophysical and pharmacological trade‐offs. Specifically, these structurally modified duplexes exhibit reduced hybridization thermodynamics (diminished Tm) compared to their unmodified native counterparts [313, 314, 315]. Furthermore, pervasive sulfur substitution drastically escalates promiscuous, non‐specific protein binding. This enhanced nonspecific association precipitates sequence‐independent off‐target effects, significantly compromising precise therapeutic target engagement and limiting the clinical utility of fully modified PS oligodeoxynucleotides [316].

To overcome the limitations of conventional modifications, Locked Nucleic Acids (LNAs)—characterized by a conformationally restricted 2′‐O,4′‐C‐methylene linkage in the ribose ring—present a highly robust structural alternative for decoy oligodeoxynucleotides (ODNs). This architectural rigidification confers unprecedented thermodynamic stability upon hybridization and robust defense against exonucleolytic degradation. Crucially, while the inherently charged phosphate backbone ensures optimal aqueous solubility and compatibility with standard delivery vectors, the incorporation of LNA monomers strictly preserves high‐affinity, specific sequestration of NF‐κB. However, maximizing the therapeutic efficacy of these modified duplexes necessitates precise spatial engineering, as the exact frequency and topological positioning of LNA residues are critical determinants for optimal NF‐κB binding [317, 318, 319, 320].

Peptide Nucleic Acids (PNAs)—uncharged, pseudo‐peptide analogs based on N‐(2‐aminoethyl) glycine—have emerged as an alternative decoy platform, conferring absolute structural resistance against both nucleolytic and proteolytic degradation [321, 322, 323]. Despite these metabolic advantages, their therapeutic utility is severely limited by binding deficiencies; pure PNA oligomers exhibit negligible sequestration capacity for transcription factors like NF‐κB. Although NF‐κB (e.g., the p52 subunit) can recognize DNA/PNA chimeras, these hybrid duplexes suffer from substantially inferior kinetic and thermodynamic stability compared to native double‐stranded DNA constructs [321]. Consequently, in a direct comparative context, Locked Nucleic Acids (LNAs) fundamentally outperform PNAs in decoy engineering. The inherently superior binding affinity and enhanced thermal stability of LNA‐modified sequences firmly establish them as the more robust and efficacious architectural choice for targeted biomedical applications [324].

The uncharged nature of the PNA backbone deprives the system of crucial electrostatic interactions required to stabilize protein‐DNA complexes [321]. For instance, robust NF‐κB sequestration is heavily dependent on non‐sequence‐specific contacts with the charged deoxyribose phosphate backbone to reinforce binding stability [325, 326]. In contrast, the seamless integration of LNA monomers into DNA sequences via standard synthesis enables precise optimization of physicochemical properties, conferring superior nuclease resistance without sacrificing target affinity [324]. Despite these structural advancements, the clinical translation of conventional DNA decoys is still impeded by hurdles such as suboptimal therapeutic efficacy, narrow dosing windows and off‐target toxicity. To circumvent these limitations, next‐generation approaches employ advanced delivery and spatiotemporal strategies, including aptamer‐mediated targeted delivery [327] and the utilization of photocaged nucleosides that strictly activate decoy function upon ultraviolet (UV) irradiation [328].

However, the clinical translation of receptor‐mediated or photo‐stimulated delivery is often hindered by in vivo applicability challenges [329]. To bypass these limitations and achieve intrinsic cellular specificity, leveraging endogenous biomarkers—such as dysregulated microRNAs—offers a robust alternative. For instance, miR‐21 can serve as an intracellular trigger within Catalytic Hairpin Assembly (CHA) systems [330]. In this targeted paradigm, disease‐specific overexpression of miRNAs catalyzes the assembly of two complementary DNA hairpins (HP1 and HP2), driving the amplified and selective generation of NF‐κB decoys exclusively within malignant cells. Furthermore, selective phosphorothioation of the hairpin toehold regions strategically confers DNase resistance while maintaining the catalytic fidelity of the reaction. This stimulus‐responsive platform enables precise, biomarker‐driven decoy regulation, paving the way for personalized therapeutic interventions in drug‐resistant pathologies [331].

Collectively, advanced bioengineering paradigms—particularly LNA integration and CHA‐mediated systems—effectively circumvent the intrinsic limitations of conventional DNA decoys. These sophisticated molecular engineering strategies are redefining the therapeutic landscape of nucleic acid therapeutics, driving their successful translation into highly precise and clinically viable modalities.

### 12.2 LNP Transporters for DNA Decoy Delivery

Nanoparticle (NP)‐mediated encapsulation has emerged as a minimally invasive and highly efficient platform for the targeted delivery of therapeutic agents. However, the clinical translation and large‐scale manufacturing of these systems are hindered by significant bioengineering bottlenecks, primarily the requisite precise calibration of particle size, surface chemistry and drug release profiles [331]. The in vivo fate and pharmacokinetic behavior of these nanocarriers are strictly governed by their physicochemical determinants—namely size, surface charge, polydispersity index (PDI), morphology and release kinetics. Ultimately, efficient cellular internalization, which is predominantly driven by concentration‐dependent endocytic pathways, directly dictates the intracellular bioavailability and the subsequent cytotoxic profile of the therapeutic payload [332].

Lipid Nanoparticles (LNPs) represent a vanguard platform for Oligonucleotide Therapeutics (ONTs), meticulously engineered through the multicomponent assembly of cationic/ionizable lipids, cholesterol, phospholipids, and PEGylated‐lipids. This sophisticated formulation confers essential payload protection against enzymatic degradation, thereby prolonging circulation time and facilitating efficient cytoplasmic release. The strategic incorporation of targeting ligands enables precise cellular tropism toward specific cell populations, such as cancer or immune cells, a strategy leveraged across various non‐viral vectors [300].

Furthermore, engineering ‘smart’ liposomal systems for stimuli‐responsive behaviour (e.g., pH or oxidative sensitivity) significantly enhances therapeutic specificity while minimizing off‐target toxicities. Collectively, the inherent biocompatibility, robust encapsulation capabilities, and nuclease resistance of these nanocarriers establish them as an ideal conduit for therapeutic agents. The ultimate efficacy and safety profile are intrinsically linked to nanoparticle concentration, which dictates the kinetics of cellular uptake via endocytic pathways and governs the subsequent cytotoxic response [332].

### 12.3 Critical Summary: Transcending the Limitations of Blunt Pharmacological Blockade

The pharmacological blockade of NF‐κB has historically been trapped in a translational bottleneck, where the potent anti‐inflammatory efficacy of conventional pan‐inhibitors (e.g., glucocorticoids) is inextricably linked to severe systemic toxicity and dose‐limiting immunosuppression. The contemporary therapeutic paradigm must pivot from generic upstream kinase inhibition toward transcriptomic precision and context‐dependent modulation. Oligonucleotide Therapeutics (ONTs) and dominant‐negative biologics (e.g., IκBα super‐repressor) offer unprecedented molecular selectivity, conceptually eliminating the off‐target promiscuity typical of small‐molecule kinase inhibitors. Parallelly, multi‐target phytochemicals present a compelling adjunctive strategy by dismantling oxidative‐inflammatory feed‐forward loops. However, the ultimate clinical viability of these advanced modalities strictly hinges on overcoming their inherent pharmacokinetic vulnerabilities, dictating an absolute requirement for next‐generation, tissue‐selective formulation strategies to widen the therapeutic index.

#### 12.3.1 Bioengineering the Optimal Transcriptional Sink

Decoy oligodeoxynucleotides (ODNs) represent an elegant ‘transcriptional sink’ strategy, circumventing the extreme redundancy of upstream signalling networks by exclusively targeting the terminal DNA‐binding event. Yet, their translation has been plagued by the classic oligonucleotide dilemma: balancing nucleolytic stability with target affinity. The failure of extensive phosphorothioate (PS) modifications, which induce catastrophic off‐target protein promiscuity, alongside the biophysical inadequacy of uncharged Peptide Nucleic Acids (PNAs), underscores the necessity for rational structural rigidification. Locked Nucleic Acids (LNAs) have unequivocally emerged as the superior architectural framework, conferring absolute nuclease resistance while strictly preserving the electrostatic integrity required for robust NF‐κB sequestration. Moving forward, the integration of these high‐affinity LNA decoys into stimulus‐responsive paradigms—such as microRNA‐driven Catalytic Hairpin Assembly (CHA) systems—marks a definitive leap toward spatiotemporal precision, enabling the autonomous, biomarker‐triggered activation of therapeutics exclusively within malignant or inflamed microenvironments.

#### 12.3.2 Bridging the Translational Divide With Smart Nanocarriers

The immense therapeutic potential of nucleic acid‐based NF‐κB modulators remains largely theoretical in the absence of sophisticated delivery architectures. Lipid Nanoparticles (LNPs) have definitively resolved the foundational pharmacokinetic challenges of oligonucleotide therapeutics, providing indispensable protection against ubiquitous nucleases while facilitating critical endosomal escape. However, the next monumental frontier in nanomedicine is achieving precise extra‐hepatic targeting and microenvironment‐specific payload release. The rational engineering of ‘smart’, stimuli‐responsive LNPs—meticulously calibrated to exploit disease‐specific pathophysiological cues such as localized acidosis or elevated reactive oxygen species (ROS)—will definitively mitigate non‐specific biodistribution. By seamlessly integrating targeted surface ligands with dynamic, responsive lipid compositions, these advanced nanocarriers will successfully transform highly potent, yet vulnerable, NF‐κB decoys into clinically viable precision medicines.

## 13 Challenges, Limitations and Future Prospects

Despite their therapeutic promise, the clinical translation of DNA decoy oligonucleotides (ODNs) is heavily impeded by intrinsic pharmacokinetic liabilities, primarily a truncated in vivo half‐life driven by rapid intra‐ and extracellular nuclease degradation. Furthermore, achieving targeted intracellular delivery—particularly to hepatic tissues—remains a formidable translational bottleneck. The lack of precise spatial control and cell‐type specificity exacerbates safety concerns; indiscriminate transcription factor sequestration (e.g., NF‐κB inhibition in healthy cells) inevitably provokes off‐target systemic toxicities. Collectively, these biopharmaceutical limitations—rapid enzymatic clearance, poor targeting efficiency and narrow therapeutic windows—constitute a critical gap that must be bridged to realize the widespread clinical adoption of ODN therapies.

Overcoming these translational barriers necessitates the development of next‐generation decoy platforms coupled with advanced nanotechnological delivery vectors. Such integration is imperative to guarantee targeted intracellular accumulation while mitigating systemic exposure. Furthermore, given the pleiotropic physiological role of NF‐κB in cellular survival, achieving exquisite tissue specificity is paramount to circumvent severe systemic toxicities. Although the competitive DNA‐binding mechanism of ODN decoys theoretically offers a more precise, tissue‐selective inhibitory profile compared to upstream kinase inhibitors, successful clinical translation must transcend simplistic molecular paradigms. Future drug development strategies must rigorously account for profound biological complexities, including intrinsic feedback loops, signalling pathway crosstalk, host microenvironment dynamics, and the establishment of reliable dose‐escalation endpoints that accurately reflect target inhibition.

Navigating the systemic toxicity concerns associated with NF‐κB inhibition necessitates rigorous pharmacokinetic‐pharmacodynamic (PK/PD) integration. Although ubiquitously active, NF‐κB orchestrates highly specific, context‐dependent transcriptional networks, providing a nuanced therapeutic window. Consequently, rational drug design must leverage this regulatory complexity. Failing to do so can precipitate paradoxical adverse effects; for instance, sustained IKKβ suppression paradoxically induces IL‐1β hypersecretion and neutrophilia secondary to protease dysregulation [82, 83]. Ultimately, mitigating inherent on‐ and off‐target clinical liabilities mandates the early implementation of robust target‐engagement biomarkers in translational strategies.

Targeting NF‐κB offers a profound strategic advantage, acting as a ‘therapeutic bottleneck’ to simultaneously attenuate pleiotropic downstream pro‐inflammatory cascades in multifaceted pathologies like NASH. Nevertheless, the clinical translation of oligonucleotide therapeutics (ONTs) against this axis is fundamentally impeded by formidable physiological barriers, primarily the vascular endothelium and extracellular matrix (ECM). Consequently, realizing the full therapeutic efficacy of ONTs necessitates the innovation of advanced carrier platforms engineered to optimize tissue penetrance and cellular delivery.

## 14 Conclusion

The therapeutic manipulation of the NF‐κB cascade, particularly via IKKβ inhibition, mandates a rigorous appraisal of the localized molecular microenvironment and genetic context. While the pathogenic interplay between the NF‐κB, MAPK and JAK–STAT axes is fundamentally established in inflammatory diseases, the non‐canonical NF‐κB signalling pathway has distinctly emerged as a critical pathological driver, notably in hepatic pathogenesis. However, leveraging this axis for precision therapeutics is currently constrained by significant mechanistic voids. Moving forward, research must pivot from the well‐characterized cytoplasmic regulators (NIK and IKKα) to decode the understudied nuclear dynamics of RelB−p52 heterodimers. Furthermore, delineating cell‐type‐specific transcriptomic repertoires, elucidating the pleiotropic cross‐talk of NIK with parallel signalling cascades, and deciphering the spatiotemporal intersection between canonical and non‐canonical NF‐κB pathways remain imperative. Resolving these intricate regulatory ambiguities will definitively bridge the gap toward engineering highly specific, next‐generation targeted therapies for inflammatory and liver‐related pathologies.

The clinical translation of highly specific NF‐κB inhibitors remains significantly hampered by dose‐limiting systemic toxicities, underscoring the absolute necessity for precision dosing paradigms. Consequently, leveraging pleiotropic natural compounds or repurposing pharmacological agents with favourable safety profiles—either as standalone therapeutics or synergistic adjuvants—offers a pragmatic alternative that warrants robust clinical validation. A formidable impediment to these translational efforts is the profound tissue‐specific heterogeneity of NF‐κB activation, compounded by the lack of direct in vivo quantification tools. Surmounting this challenge necessitates the operationalization of surrogate biomarkers, such as free light chains (FLCs), to dynamically monitor target engagement and therapeutic efficacy, particularly in cardiometabolic syndromes. Ultimately, comprehensively mapping the NF‐κB regulatory networks will catalyse the development of advanced diagnostic and therapeutic strategies across diverse inflammation‐driven pathologies, encompassing autoimmune, oncological, and neurodegenerative disorders. Crucially, amidst the escalating global burden of refractory hepatic diseases, targeting interconnected immunometabolic hubs—most notably the SIRT1/NF‐κB axis—represents an unprecedented and highly promising frontier for definitive clinical breakthroughs.

### 14.1 The Paradigm Shift in NF‐κB Targeted Therapeutics

Despite decades of rigorous mechanistic elucidation, the translational trajectory of NF‐κB‐targeted interventions remains fundamentally bottlenecked by the conventional paradigm of global pathway suppression, which invariably provokes systemic toxicity and disrupts basal immune homeostasis. The critical imperative for future research is a decisive departure from blunt molecular antagonism toward spatiotemporally precise, context‐aware modulation. This evolution mandates the integration of advanced targeted delivery platforms with a systems‐biology understanding of pleiotropic immunometabolic hubs, particularly the SIRT1/NF‐κB axis. Furthermore, bridging the translational chasm requires abandoning static preclinical endpoints in favour of dynamic in vivo profiling, leveraging surrogate biomarkers to ensure real‐time target engagement without compromising safety. Ultimately, realizing the clinical potential of NF‐κB modulation—especially in refractory hepatic pathologies—hinges on decoding the nuanced cross‐talk between its canonical and non‐canonical arms within specific cellular microenvironments, thereby transforming a complex, intractable biological network into a druggable precision target.

## Author Contributions

**Bherouz Pourdad:** conceptualization, investigation, formal analysis, writing – original draft, writing – review and editing, supervision, data curation. **Arash Pourdad:** data curation, conceptualization, investigation, writing – original draft.

## Funding

The authors have nothing to report.

## Conflicts of Interest

The authors declare no conflicts of interest.

## Data Availability

Data sharing is not applicable to this article as no new data were created or analysed in this study.
